# Unveiling the “Less is More” paradox: How experience and cognitive filling drive attractiveness in occluded faces

**DOI:** 10.1186/s41235-025-00691-w

**Published:** 2025-11-27

**Authors:** Yurou Gao, Mengliang Cao, Ruoying Zheng, Guomei Zhou

**Affiliations:** Department of Psychology, Sun Yat-sen University, No. 132, Wai Huan East Road, Higher Education Mega Center, Panyu District, Guangzhou, 510006 Guangdong China

**Keywords:** Less is More, Facial attractiveness, Occlusion, Information shortage, COVID-19, Experience, Familiarity, Average face, Average/typical filling hypothesis, Matching filling hypothesis

## Abstract

The “Less is More” phenomenon, the intriguing elevation of facial attractiveness under conditions of limited visual information (e.g., masked faces), has garnered significant attention since the COVID-19 pandemic. This study delves into the influence of experience on this phenomenon and its underlying cognitive mechanisms through three experiments. Experiment 1 examined attractiveness ratings of occluded, predicted and complete faces with four occlusion types before and after the pandemic onset. Experiment 2 investigated how participants mentally complete faces occluded by sunglasses by selecting from high-, low-attractiveness, and average eye representations. Experiment 3 explored the impact of familiarity. Participants first familiarized themselves with half of the faces, then rated the attractiveness of the occluded and predicted versions of all familiar and unfamiliar faces, and finally chose the original complete face from a set of foils (high-attractiveness, low-attractiveness, and average face) . Across all experiments, the inherent attractiveness of the complete faces was manipulated. Our findings reveal a clear effect of experience on the “Less is More” effect: it was amplified post-pandemic (Experiment 1), more pronounced for unfamiliar faces (Experiment 3), and consistently stronger for initially low-attractiveness faces. Selection patterns in Experiments 2 and 3, with average faces chosen most often for unfamiliar faces, support the Average/Typical Filling hypothesis. However, familiar faces elicited a preference for the original features, suggesting a memory-driven process. These results revealed a dynamic interplay between prior experience and visual constraints in shaping facial attractiveness perception.

**Statement of significance:** The ubiquitous adoption of face masks during the COVID-19 pandemic, a global reality that suddenly obscured portions of the face, provided an unprecedented lens through which to examine the fundamental principles of facial attractiveness perception. The intriguing “Less is More” effect, where masked faces often appeared more appealing, directly inspired our investigation into how the human brain processes incomplete visual information and the role of experience in shaping these judgments. By systematically analyzing attractiveness ratings across diverse occlusion types before and after the pandemic and by exploring the cognitive “filling-in” mechanisms for both familiar and unfamiliar individuals, our work transcends a mere description of the “mask effect”. Instead, it delves into the core processes underlying attractiveness evaluation when visual input is limited. Our findings reveal that the “Less is More” effect was amplified by increased real-world exposure to masked faces, and that our mental reconstructions of occluded faces diverge based on familiarity. Importantly, our results also identify specific occlusion types that most effectively enhance perceived attractiveness. This “use-inspired basic research,” born from a tangible global event, not only illuminates the psychological impact of the pandemic but also yields fundamental insights into the adaptable nature of attractiveness perception under conditions of limited visual information, with potential relevance for understanding aesthetic preferences in various real-world contexts, including virtual interactions and artistic design.

**Supplementary Information:**

The online version contains supplementary material available at 10.1186/s41235-025-00691-w.

The COVID-19 pandemic necessitated the widespread use of face masks, forcing us to rely on incomplete visual cues to make social judgments, such as facial identity, emotion, age, and attractiveness. This real-world challenge has driven scientists to study how we make these judgments with limited visual input on identity (e.g., Carragher & Hancock, 2020; Stajduhar et al., 2022), emotion (Blazhenkova et al., 2022; Primbs et al., 2022), age (Ganel & Goodale, 2022; Thorley et al., 2022), and attractiveness (Pazhoohi & Kingstone, 2022). While emerging studies have begun to explore the attractiveness of occluded faces, the cognitive mechanisms underlying such judgments remain insufficient understood. The present study aims to fill this gap.

Strong evidence shows that people can judge facial attractiveness even with limited visual information. For instance, people can differentiate between faces of varying attractiveness when faces are masked or cropped (Pazhoohi & Kingstone, 2022), presented extremely briefly (e.g., 13 ms; Olson & Marshuetz, 2005), or displayed in low resolution (Bachmann, 2007). Notably, the eye’s attractiveness strongly predicts overall facial attractiveness (Gao et al., 2025; Liu et al., 2022; Saegusa & Watanabe, 2016; Santos & Young, 2011), suggesting that we can rely on key features to infer attractiveness when full facial information is unavailable.

However, two critical questions remain unresolved regarding the perception of partial faces: (1) Do faces with partial visual information lead to consistently higher or lower attractiveness ratings compared to fully visible faces? Existing findings remain inconsistent. (2) What mental processes underlie the reconstruction of attractiveness judgments from fragmented perceptual input? These unanswered questions highlight the need to investigate not only whether partial visual information *enhances or diminishes* attractiveness, but also to uncover the precise cognitive mechanisms at play.

## Less is more?

Numerous studies have found that facial attractiveness can be enhanced under conditions of limited information compared to complete faces. Here, we refer it as “Less is More” effect. For example, attractiveness may increase when faces are downsized, blurred, or subjected to mosaic processing (Orghian & Hidalgo, 2020), when spatial frequency and contrast of the faces are reduced, or when part of the face is occluded (Sadr & Krowicki, 2019), when only portions of faces are presented (Gao et al., 2025; Liu et al., 2022; Orghian & Hidalgo, 2020). However, the impact of occlusion on facial attractiveness can be influenced by the type of occlusion, the inherent attractiveness of the face, and the prior experience.

### Effect of the type of occlusion and the inherent attractiveness of the face

The “Less is More” effect, which suggests that occlusion can sometimes enhance attractiveness, depends on the interaction between occlusion type and inherent attractiveness. Miyazaki and Kawahara (2016) found that covering the lower half of the face with a notebook increased the attractiveness of less attractive faces by concealing flaws, but decreased the attractiveness of highly attractive faces by obscuring their desirable features. In contrast, when the same region was covered by a sanitary mask, attractiveness ratings consistently decreased across all levels of inherent facial attractiveness (Miyazaki & Kawahara, 2016).

However, subsequent research by Kawahara and his colleagues (Kamatani et al., 2021) provided a more nuanced picture regarding masks, replicating the decrease in attractiveness for masked highly attractive faces; crucially, Kamatani et al. (2021) also found that mask-wearing increased the perceived attractiveness for low-attractive faces, while having no significant effect for faces with moderate attractiveness scores.

A similar interaction between facial occlusion and inherent attractiveness was observed when using transparent masks. Lee and Jeong (2023) found that transparent masks primarily benefit less attractive faces, while diminishing the attractiveness of highly attractive individuals. This effect may be attributed to the distortion that transparent masks can cause to lower facial features, which play a crucial role in attractiveness judgments.

In line with these findings, several other studies have demonstrated that the “Less is More” effect is more pronounced for less attractive faces when wearing masks (e.g., Hewer & Lewis, 2024; Jeong et al., 2023; Pazhoohi & Kingstone, 2022). However, the effect on highly attractive faces is mixed. For instance, Pazhoohi and Kingstone (2022) observed no significant effect of cloth masks on the attractiveness of highly attractive faces, while Hies and Lewis (2022) found that sanitary masks, cloth masks, and notebooks could enhance the attractiveness of these faces. Notably, studies that did not directly manipulate facial attractiveness have also yielded inconsistent results regarding the impact of sanitary masks (increase in attractiveness, Lau, 2021; no effect, Guo et al., 2022), possibly due to variations in the inherent attractiveness levels of the faces used.

Taken together, these inconsistencies highlight the need for further research to elucidate the complex interplay of factors influencing the “Less is More” effect under conditions of facial occlusion.

### Effect of experience on “Less is More”

Experience with occluded faces likely modulates the “Less is More” effect. Evidence for this comes from contrasting findings observed before and after the widespread adoption of mask-wearing. Miyazaki and Kawahara (2016) suggested that the negative effect of sanitary masks on both low- and high-attractiveness faces was potentially due to an association between masks and illness (Jones et al., 2004). However, Kamatani et al. (2021) found that as mask-wearing became normalized during the COVID-19 pandemic, its impact on low-attractiveness faces shifted from negative to positive. This suggests that the initial negative perception of sanitary masks, potentially acting as a “prime of unhealthiness,” has weakened over time due to habituation and a change in their social meaning, from a signal of illness to a symbol of social compliance (Nakayachi et al., 2020). This aligns with the phenomenon where frequent exposure to an object can increase positive evaluations (Courtice et al., 2023; Dudarev et al., 2022a, 2022b). Moreover, individuals who support mask-wearing tend to perceive masked faces more favorably (Dudarev et al., 2022a, 2022b). These temporal shifts and individual inferences in the perception of masked faces illustrate that experience can influence the “Less is More” effect in the context of facial occlusion.

The impact of mask-wearing on facial attractiveness also varies across racial groups, potentially reflecting different experiences with (masked) faces of various races. Dudarev et al., (2022a, 2022b) found that same-race (White European) individuals wearing masks were generally perceived as more attractive than their unmasked counterparts, while masked other-race (Asian) individuals were rated as less attractive than unmasked individuals. Furthermore, Kamatani et al. (2023) observed that wearing sanitary masks reduced the facial attractiveness of high-attractiveness faces within the same race (Japanese Asian). However, for individuals of different races (White European, Black) or different groups (non-Japanese Asian), regardless of the initial attractiveness level of the face, masks tended to increase facial attractiveness evaluations. These findings suggest a potential in-group bias in the perception of masked faces, where familiarity and experience with (masked) faces of one’s own racial group might lead to different evaluations compared to those of other racial groups.

### How do people process facial attractiveness with limited information?

Beyond the occlusion account, which attributes the effect to concealing flaws or obscuring desirable features, and the unhealthiness priming hypothesis proposed by Miyazaki and Kawahara (2016), several mental completion hypotheses have been put forward to explain the “Less is More” phenomenon, proposing how observers infer the missing facial information from partially visible faces.

**The Most Beautiful Filling.** Sadr and Krowicki (2019) proposed an evolutionary perspective, suggesting that when visual information is incomplete, observers may default to mentally reconstructing idealized, highly attractive features. However, this hypothesis currently lacks empirical support.

**Average/Typical Filling.** Orghian and Hidalgo (2020) hypothesized that we use an ‘archetype’ representation, or an average template, to fill in missing facial information. Kramer and Jones (2022) provided empirical support for this hypothesis. In their study, participants viewed the upper or lower half of faces and adjusted a continuously knob to modify the averageness of the implied missing parts. The results indicated that participants consistently selected representations that were more average than the original faces, providing direct evidence for the use of average/typical internal representations when inferring faces with limited information. However, these findings may be difficult to distinguish from the most beautiful filling hypothesis because average faces are often considered more attractive than individual original faces (Langlois & Roggman, 1990).

**Moderate Attractiveness Filling**. Kamatani et al. (2023) proposed that people use a representation of moderate attractiveness, rather than an average one, to infer own-race occluded faces. This explains why sanitary masks reduced the facial attractiveness of high-attractiveness faces within the same race (Japanese Asian). In contrast, other-race (White European, Black) faces or different-group (non-Japanese Asian) faces may lack of well-established counterpart templates from accumulated experience. As a result, the template used for other-race faces might be biased toward highly attractive exemplars (e.g., celebrities), leading to an increase in attractiveness ratings regardless of inherent attractiveness. However, this hypothesis lacks direct empirical support and does not explain the finding by Dudarev et al., (2022a, 2022b) where masked other-race (Asian) faces were rated as less attractive than unmasked other-race faces.

### Current study

Despite growing interest in facial attractiveness perception with partial visual information, two critical questions remain unresolved: (1) Do faces with partial visual information consistently elicit different attractiveness ratings compared to fully visible faces? (2) What mental processes underlie the reconstruction of attractiveness judgments from fragmented perceptual input? Furthermore, the role of experience and the underlying mechanisms of the “Less is More” phenomenon are underexplored. For instance, it is unknown whether the potential impact of the COVID-19 pandemic (i.e., increased experience with mask-occluded faces) on this effect generalizes to other types of occlusions, such as sunglasses or hands. It is also unclear whether the effect differs between familiar and unfamiliar faces, and whether the underlying cognitive mechanisms are consistent across these conditions.

It is also worth noting the lack of consensus in previous research regarding the assessment of the “Less is More” effect. Some studies have compared the attractiveness of occluded faces directly to that of complete faces (e.g., Lee & Jeong, 2023; Pazhoohi & Kingstone, 2022), while others have examined how participants’ predictions based on the occluded portion match the actual attractiveness of the complete faces (e.g., Mihara et al., 2023).

**Fig. 1:**
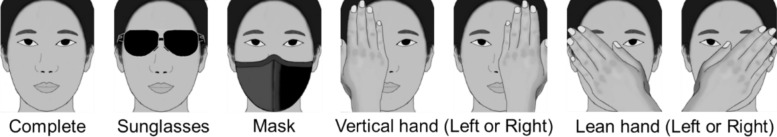
Example of stimuli in experiment 1. *Note.* The formal experiment utilized real facial photographs. Due to copyright restrictions, caricatures are used for illustration purpose here

To address these questions, our study investigates the role of experience and the underlying mechanisms of the “Less is more” phenomenon across three experiments. Experiment 1 examined changes in the “Less is more” phenomenon before (2019 April) and after (2022) the COVID-19 pandemic. Participants viewed four types of natural occlusions (sunglasses, mask, vertical hand, and lean hand; see Fig. 1). For each face, they rated the attractiveness of occluded faces, predicted the attractiveness of the complete face, and then rated the attractiveness of the actual complete face. Experiment 2 investigated the underlying mechanism by asking participants to choose one of three eye representations (high-attractiveness, low-attractiveness, or average) to mentally complete a face occluded by sunglasses. In Experiment 3, we further explored the role of familiarity by having participants rate the attractiveness of occluded, predicted, and complete versions of both familiar and unfamiliar faces. Subsequently, they chose one of four eye representations (high-attractiveness, low-attractiveness, average, or the original eyes) to complete a face occluded by sunglasses. Across all three experiments, the inherent attractiveness of the original complete faces was systematically manipulated to examine whether the “Less is More” effect is specific to low-attractiveness faces or generalizable across all levels of facial attractiveness.

## Experiment 1: “Less is More” before and after the COVID-19 pandemic

Experiment 1 examined the “Less is More” effect when low-/high-attractiveness faces were naturally occluded before (April 2019, Experiment 1a) and after (2022, Experiment 1b) the COVID-19 pandemic.

Guided by the principle that experience shapes attitudes toward objects (Dudarev et al., 2022a, 2022b) and acknowledging the shift effect of masks—from consistently decreasing attractiveness before COVID-19 (Miyazaki & Kawahara, 2016) to increasing it for low attractiveness faces after COVID-19 (Kamatani et al., 2021)—we hypothesized specific effects for 2022. We predicted a more pronounced “Less is More” effect, particularly for mask occlusion due to its pandemic-related prevalence. Furthermore, we expected that this phenomenon to be stronger for inherently low-attractiveness faces than for high-attractiveness faces.

## Method

### Participants

We conducted a power analysis using Power ANalysis for GEneral Anova designs (PANGEA; Westfall, 2016) to determine the required sample size. A sample size of 14 was required to reveal a medium effect (*d* = 0.45) of 2 (Year) × 2 (Inherent attractiveness) × 5 (Occlusion) interaction with power of 0.825 and Replicates = 20, var (error) = 0.2, var (Participants × Inherent attractiveness × Occlusion) = 0.08 as default in a 2 (Year) × 2 (Inherent attractiveness) × 2 (Task) × 5 (Occlusion) mixed-design ANOVA for Experiment 1. Nevertheless, we increased the sample size to 32 in each sub-experiment to ensure sufficient statistical power.

In total, 64 undergraduate students from XXX University participated in Experiment 1. Thirty-two of them (16 females, 16 males; *M*_age_ = 19.66 years, SD_age_ = 1.68 years) participated in Experiment 1a in 2019 and thirty-two of them (16 females, 16 males; *M*_age_ = 20.72 years, SD_age_ = 2.22 years) participated in Experiment 1b in 2022. All participants provided informed consent before participating and received compensation for their time after the experiment. They reported having normal or corrected-to-normal vision and were unfamiliar with the faces used in the experiment.

### Stimuli

We used the twenty high-attractive and twenty low-attractive greyscale Chinese-face morphed photographs from Wang et al. (2015). As detailed in that study, each high-attractive face was created by morphing 55% of a celebrity’s face with 45% of an ordinary face. Similarly, each low-attractive face was created from a blend of 15% celebrity and 85% ordinary face. The two levels share subtle physical features, which minimizes the influence of facial metrics on attractiveness ratings. Additionally, neither face is recognizable as a specific celebrity, which controls for the confounding effect of familiarity. According to the original study by Wang et al. (2015), the female and male faces were independently rated by 30 opposite-sex participants on a 9-point Likert scale (where 1 = *very unattractive* and 9 = *very attractive*). High-attractive faces: 10 males, *M* = 4.93, *SD* = 0.20; 10 females, *M* = 4.58, *SD* = 0.20. Low-attractive faces: 10 males: *M* = 2.57, *SD* = 0.18; 10 females: *M* = 2.96, *SD* = 0.18. Paired-samples *t* tests confirmed significant differences [male: *t*(29) = 12.94, *p* < .001; female, *t*(29) = 15.54, *p* < .001].

Each original (complete version) face was digitally manipulated to create four occluded versions: sunglasses, mask, vertical hand (left and right sides), and lean hand (left and right sides) (see Fig. 1), resulting in a total of 280 stimuli (40 original faces × 7 versions).

### Procedure

**Fig. 2:**
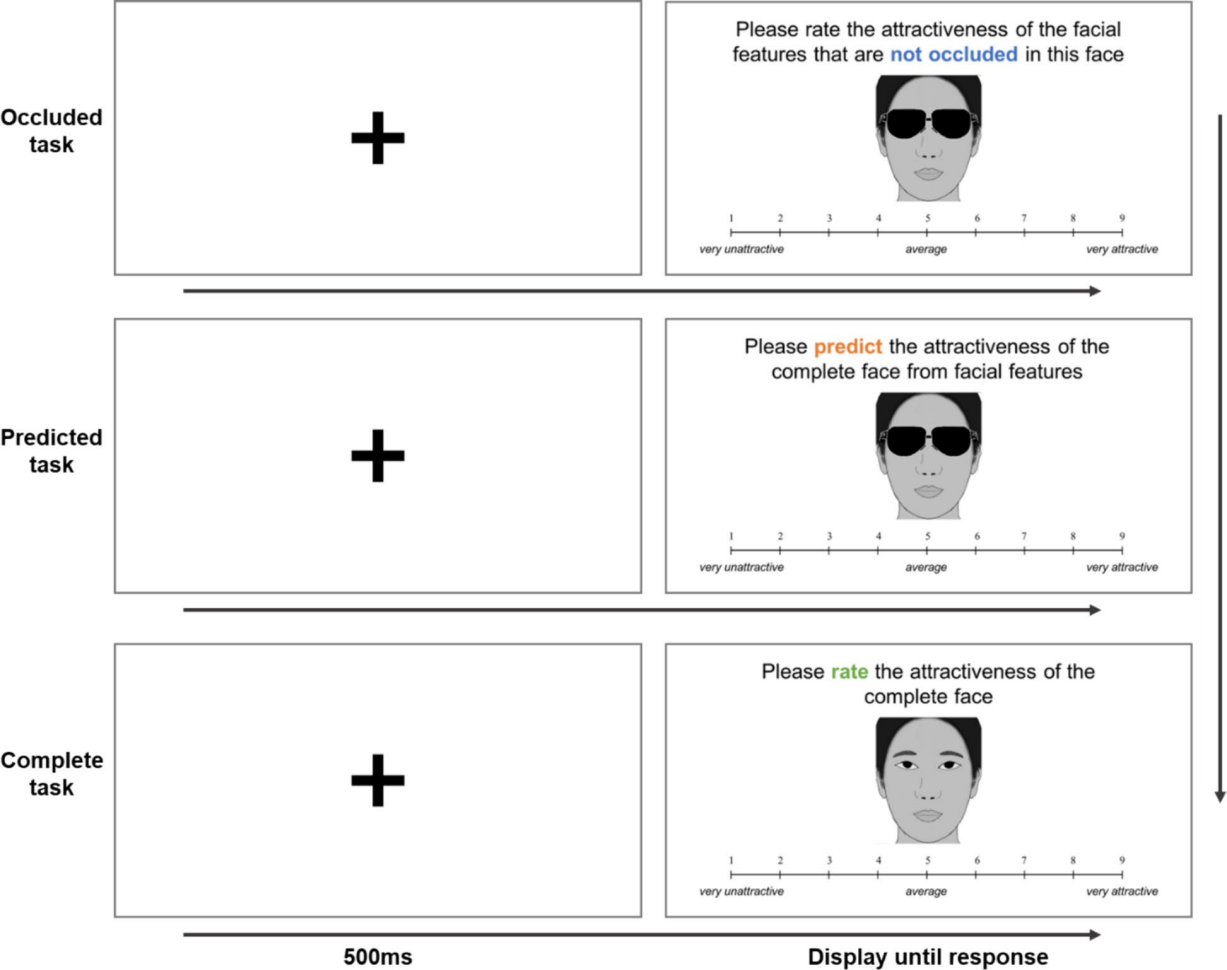
The procedure illustration of experiment 1

The experiment consisted of three sequential blocks, each corresponding to a distinct task (see Fig. 2). **Occluded Task**: Participants rated the attractiveness of the visible part of the face. **Predicted Task**: Participants predicted the attractiveness of the complete face based on the visible part of the face. **Complete Task**: Participants rated the attractiveness of the original complete faces. The first two blocks (Occluded and Predicted) were further divided into four groups, each corresponding to a different occluded version: sunglasses, mask, vertical hand, and lean hand. The Complete Task block consisted of a single group. Each group contained 40 trials, with faces presented in a random order.

Each trial began with a 500 ms fixation cross presented at the center of the screen. Subsequently, a face stimulus (either occluded or complete) was displayed centrally. Below the stimulus, a 9-point Likert scale was presented for participants to rate the facial attractiveness (1 = *very unattractive,* 5 = *average,* 9 = *very attractive*). Participants were allowed to respond at their own pace without time constraints.

The experiment was programed using E-Prime 2.0 and conducted on a 23-inch CRT monitor with a resolution of 1920 × 1080 pixels and a refresh rate of 60 Hz.

### Data analysis

Data from all 64 participants were included in the analyses. The attractiveness ratings for the left and right vertical hand occlusions were averaged to represent the vertical hand condition. A similar approach was used for the lean hand condition. To test the assumption of sphericity, Mauchly’s test was conducted. When the assumption of sphericity was violated, degrees of freedom were adjusted using the Greenhouse–Geisser correction. Statistical significance was determined accordingly. Effect sizes were estimated using $\eta_{p}^{2}$. For significant interactions, simple main effect analyses were conducted to further explore the impact of different manipulations on the dependent variable. Post hoc comparisons were conducted using the Bonferroni correction, and effect sizes were estimated using Cohen’s* d*.

## Results and discussion

### The “Less is More” effect across different occlusion types

Since the levels of Occlusion does not apply to the Complete Task, this Task was treated as a separate level within the Occlusion factor (i.e., complete face) for the purposes of analysis. A 2 (Year: 2019, 2022) × 2 (Inherent attractiveness: high, low) × 2 (Task: Occluded, Predicted) × 5 (Occlusion: complete face, sunglasses, mask, vertical hand, lean hand) mixed-design ANOVA was conducted on attractiveness ratings with Year as between-subjects factor, and all other variables as within-subjects factors (see Table 1 for the results).

**Table 1:** Results of 2 (Year) × 2 (Inherent Attractiveness) × 2 (Task) × 5 (Occlusion) Mixed-design ANOVA in Experiment 1

Effect	*F*	*df*	*p*	$\eta_{p}^{2}$
Year (Y)	**7.083**	**(1, 62)**	**.010**	**.103**
Inherent Attractiveness (A)	**447.409**	**(1, 62)**	** < .001**	**.878**
Task (T)	**5.335**	**(1, 62)**	**.024**	**.079**
Occlusion (O)	**34.585**	**(3.48, 215.72)**	** < .001**	**.358**
Y × A	0.795	(1, 62)	.376	.013
Y × T	0.897	(1, 62)	.347	.014
Y × O	2.324	(3.48, 215.72)	.067	.036
A × T	**7.480**	**(1, 62)**	**.008**	**.108**
A × O	**18.697**	**(3.32, 206.06)**	** < .001**	**.232**
T × O	1.803	(3.33, 206.57)	.141	.028
Y × A × T	0.048	(1, 62)	.828	.001
Y × A × O	**3.366**	**(3.32, 206.06)**	**.016**	**.051**
Y × T × O	0.872	(3.33, 206.57)	.466	.014
A × T × O	1.750	(3.30, 204.07)	.153	.027
Y × A × T × O	0.426	(3.30, 204.07)	.752	.007

Bold indicates significant effects

A significant main effect of Facial Attractiveness was found, with high-attractiveness faces (*M* = 5.968, *SD* = 0.083) rated higher than low-attractiveness faces (*M* = 4.159, *SD* = 0.106), confirming the validity of our stimulus selection.

Consistent with the “Less is More” effect, we observed a main effect of Occlusion, with attractiveness ratings significantly higher for faces with four types of occlusions (*M*_sunglasses_ = 4.958, *SD* = 0.098; *M*_mask_ = 5.442, *SD* = 0.092; *M*_vertical_ = 4.947, *SD* = 0.094; *M*_lean_ = 5.181, *SD* = 0.092) compared to complete faces (*M* = 4.787, *SD* = 0.090; *ps* < .031).

**Fig. 3:**
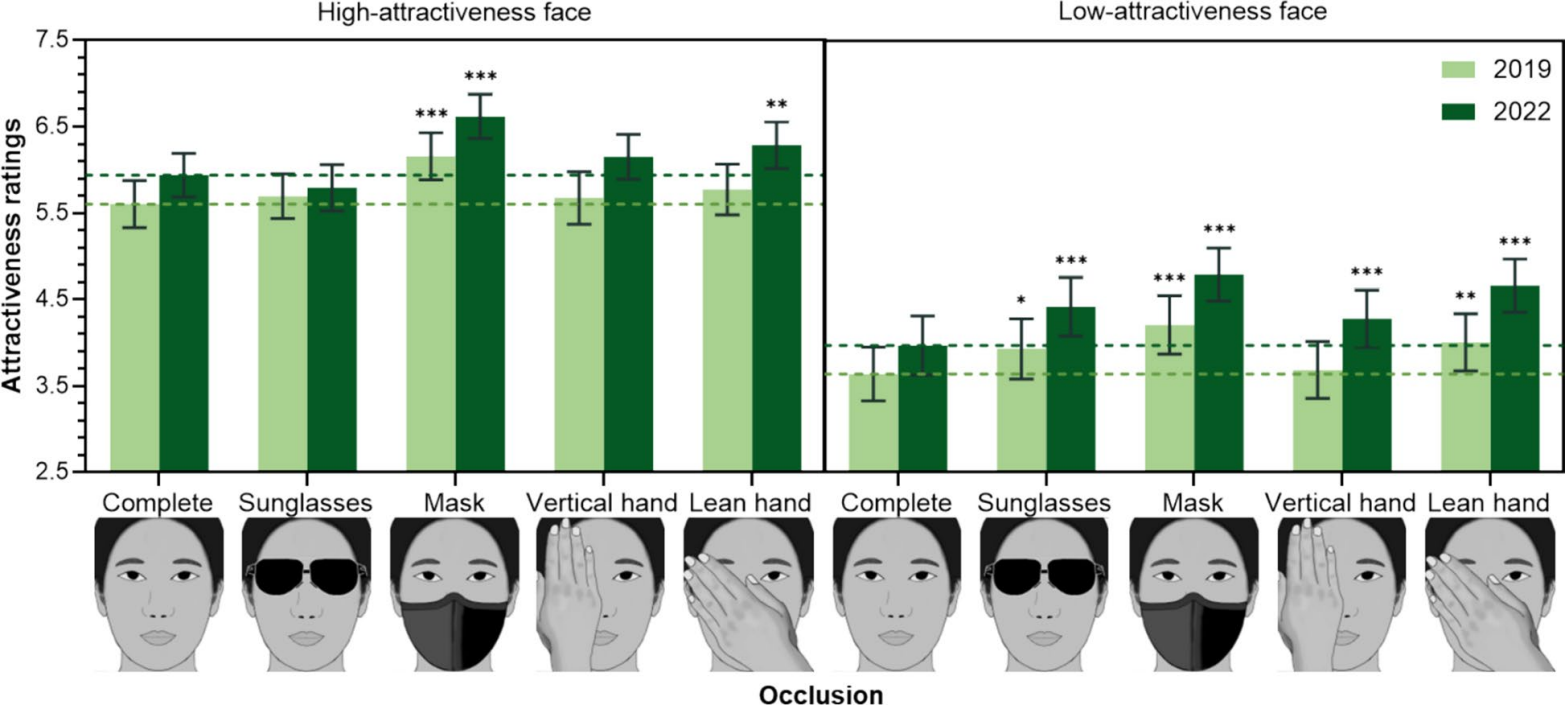
The Three-way Interaction of Occlusion × Inherent attractiveness × Year in Experiment 1. *Note.* The error bars represent the 95% confidence interval. The dashed line marks the attractiveness ratings of complete faces under different conditions. *Indicates the significance of the attractiveness ratings of occluded faces compared to the complete faces (*p =  < .05, **p =  < .01, ***p =  < .001)

The effect of Occlusion was significantly modulated by Inherent Attractiveness, and this interaction was further influenced by Year. Although separated 2 (Inherent attractiveness) × 5 (Occlusion) ANOVAs for 2019 and 2022 both revealed significant Occlusion × Inherent attractiveness interactions (see Fig. 3; for detailed results, see Table S1 in the Supplementary Information), the specific patterns differed.

In 2019, for high-attractiveness faces, only mask occlusion significantly increased attractiveness compared to complete face (*p* < .001). Conversely, for low-attractiveness faces, sunglasses, mask, and lean hand occlusions all significantly increased attractiveness (*ps* < .05). In 2022, for high-attractiveness faces, mask and lean hand occlusions significantly increased attractiveness compared to complete face (*ps* < .01). However, for low-attractiveness faces in 2022, all occluded faces were rated significantly higher than the complete face (*ps* < .001). Importantly, the Inherent attractiveness × Occlusion × Year interaction was not modulated by Task, indicating that these increases in perceived attractiveness were evident in both the Occluded Task (judging the visible facial part) and the Predicted Task (predicting whole-face attractiveness).

These results highlight the differential effects of various occlusions in enhancing perceived facial attractiveness. Notably, mask occlusion, which typically leaves the eyes visible, consistently enhanced attractiveness ratings. This aligns with post-COVID-19 research showing increased attractiveness for mask-occluded faces after (e.g., Liu et al., 2022; Pazhoohi & Kingstone, 2022). This contrasts with pre-COVID-19 findings by Miyazaki and Kawahara (2016), where sanitary masks consistently decreased attractiveness. A possible explanation for this discrepancy lies in the type of mask used: Miyazaki and Kawahara (2016) used a sanitary mask, whereas our study employed a normal black cloth mask.

Beyond masks, other forms of occlusion also boosted attractiveness, albeit to a lesser degree. Vertical hand covering (*M* = 4.947) and sunglasses (*M* = 4.958) also increased attractiveness compared to complete faces (*M* = 4.787), a trend similar to that reported by Liu et al. (2022). Mask occlusion (*M* = 5.442) showed the most substantial enhancement. Interestingly, we also found that a lean hand (*M* = 5.181), a common photography pose, effectively increases attractiveness.

The impact of sunglasses on attractiveness appears to be nuanced. While sunglasses did not alter the perceived attractiveness of high-attractiveness faces, they *did* increase the attractiveness of low-attractiveness faces. This suggests that when eyes are occluded, viewers might imagine more attractive eyes, leading to an overall attractiveness boost, particularly when the original eyes are less appealing. For already highly attractive faces, the imagined eyes may simply match the existing high attractiveness, resulting in no net increase.

### The “Less is More” effect: modulation by year or inherent attractiveness

To further directly test the changes of “Less is More” effect between 2019 and 2022 for high- or low-attractiveness faces, a 2 (Year: 2019, 2022) × 2 (Inherent attractiveness: high, low) × 3 (Task: Occluded, Predict, Complete) mixed-design ANOVA was conducted. Here, Complete was analyzed as a level of Task. Additionally, the attractiveness ratings for Occluded Task and Predict Task were calculated by averaging across their four occluded versions (sunglasses, mask, vertical hand, and lean hand) separately.

**Fig. 4:**
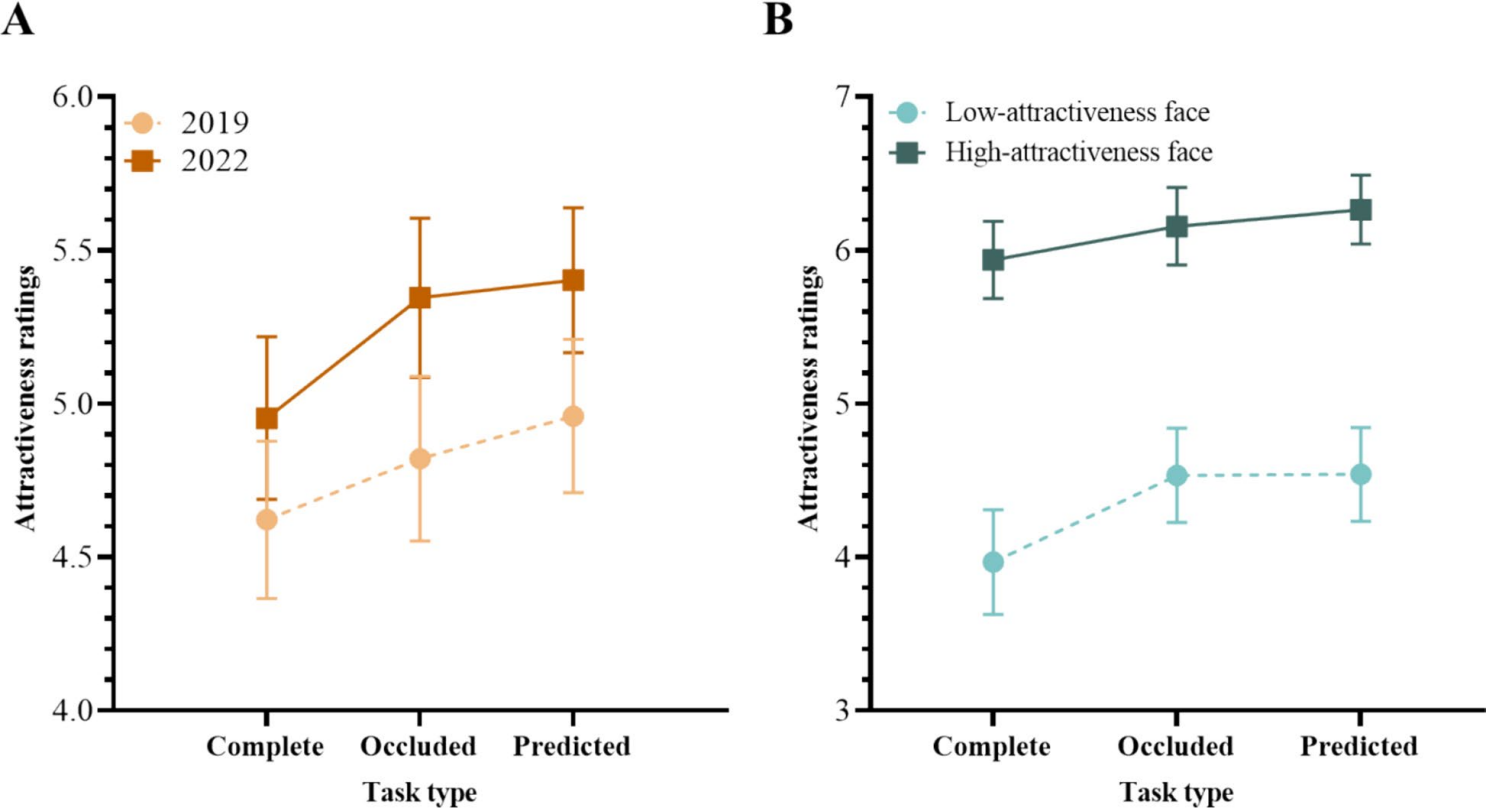
The Two-way Interaction of Task × Year and that of Task × Inherent attractiveness in Experiment 1. *Note.* The error bars represent the 95% confidence interval

Our analysis (see Fig. 4) revealed significant main effects of the three independent variables [Year: *F*(1,62) = 6.439, *p* = .014, $\eta_{p}^{2}$ = .094; Inherent attractiveness: *F*(1,62) = 455.300, *p* < .001, $\eta_{p}^{2}$ = .880; Task: *F*(2,124) = 46.070, *p* < .001, $\eta_{p}^{2}$ = .426]. We also observed a tendency for the main effect of Task to be modulated by Year [*F*(2,124) = 2.529, *p* = .084, $\eta_{p}^{2}$ = .039]. Furthermore, the interaction between Task and Facial Attractiveness was significant [*F*(1.734,107.493) = 11.589, *p* < .001, $\eta_{p}^{2}$ = .157].

The simple effect tests of the interaction tendency between Task and Year indicated consistent patterns across both years, with some nuanced differences. In 2019, both the Occluded [*t*(31) = 3.318, *p*_bonf_ = .018, Cohen’s* d* = 0.251] and Predicted [*t*(31) = 5.596, *p*_bonf_ < .001, *Cohen*’*s d* = 0.424] tasks increased the attractiveness ratings of these faces compared to the Complete task (*M* = 4.621, SD = 0.712). There was no significant difference in attractiveness ratings between the Occluded (*M* = 4.822, SD = 0.744) and Predicted tasks (*M* = 4.959, SD = 0.693) [*t*(31) = 2.278, *p*_bonf_ = .367, *Cohen*’*s d* = 0.172]. In 2022, ratings for the Occluded task (*M* = 5.345, SD = 0.720) and the Predicted task (*M* = 5.403, SD = 0.656) were again significantly higher than for the Complete task (*M* = 4.953, SD = 0.735) [Occluded vs. Complete: *t*(31) = 6.485, *p*_bonf_ < .001, *Cohen*’*s d* = 0.491; Predicted vs. Complete: *t*(31) = 7.438, *p*_bonf_ < .001, *Cohen*’*s d* = 0.563]. Similar to 2019, no significant difference was found between the Occluded and Predicted tasks [*t*(31) = 0.953, *p*_bonf_ = 1.000, *Cohen*’*s d* = 0.072].

Although the fundamental “Less is More” effect (occluded/predicted being more attractive than complete) was consistent across both years, the interaction tendency between Task and Year suggests a more pronounced “Less is More” effect in 2022. The lack of a statistically significant interaction might be due to the presence of highly attractive faces in the stimuli. Previous research indicates that masks reduced the attractiveness of highly attractive faces both before (Miyazaki & Kawahara, 2016) and after the pandemic (Kamatani et al., 2021). However, masks increased the attractiveness of less attractive faces post-pandemic (Kamatani et al., 2021). This temporal increase might be related to mask usage, as individuals who use masks more frequently tend to rate the attractiveness of masked faces higher (Dudarev et al., 2022a, 2022b).

The simple effect test of the interaction between Task and Inherent attractiveness showed that for faces with low attractiveness, ratings for the Occluded task (*M* = 4.226, SD = 0.912) and the Predicted task (*M* = 4.268, SD = 0.916) were essentially the same [*t*(31) = 0.827, *p*_bonf_ = 1.000, *Cohen*’*s d* = 0.052], and both were significantly higher than Complete tasks(*M* = 3.805, SD = 0.911) [Occluded vs. Complete: *t*(31) = 8.420, *p*_bonf_ < .001, *Cohen*’*s d* = 0.528; Predicted vs. Complete: *t*(31) = 9.247, *p*_bonf_ < .001, *Cohen*’*s d* = 0.580]. For faces with high attractiveness, ratings for the Predicted task (*M* = 6.094, SD = 0.670) were significantly higher than the Occluded task (*M* = 5.940, SD = 0.755) [*t*(31) = 3.073, *p*_bonf_ = .036, *Cohen*’*s d* = 0.193], and both were significantly higher than the Complete task (*M* = 5.770, SD = 0.741) [Occluded vs. Complete: *t*(31) = 3.413, *p*_bonf_ = .012, *Cohen*’*s d* = 0.214; Predicted vs. Complete: *t*(31) = 6.486, *p*_bonf_ < .001, *Cohen*’*s d* = 0.407]. The “less is more” effect was evident in both the Occluded and Predicted tasks. This interaction between Task and Inherent attractiveness indicated the “Less is More” effect was more pronounced for low-attractiveness faces, consistent with previous research (e.g., Hies & Lewis, 2022; Jeong et al., 2023; Kamatani et al., 2021; Lee & Jeong, 2023; Pazhoohi & Kingstone, 2022).

We also explored potential gender differences (see Hewer & Lewis, 2024) by including both face gender and participant gender as independent variables in the analysis. However, the selected complete female faces were generally rated as more attractive than the complete male faces, particularly in the high-attractiveness condition. Since attractiveness is known to influence the effect of occlusion, and both year and attractiveness can modulate the effect of occlusion (see Table 1), it is difficult to isolate whether the observed results about face gender are driven by face gender per se or by the higher attractiveness of female faces. Furthermore, due to the relatively small number of participants within each gender group, the results regarding gender differences are presented as exploratory findings in Supplementary Information rather than in the main text.

### Top versus bottom half: changing predictors of attractiveness

To examine the predictive value of specific facial regions on perceived attractiveness, we conducted stepwise multiple regression analyses separately for 2019 and 2022. In the first set of analysis (results in Table 2), attractiveness ratings from the mask condition (revealing the top half of the face) and the sunglasses condition (revealing the bottom half of the face) in the Occluded Task were used to predict attractiveness ratings in both the Predicted Task and the Complete Task. To further explore the influence of different occlusions, we conducted additional stepwise multiple regression analyses using attractiveness ratings from all four occlusion conditions (sunglasses, mask, vertical hand, and lean hand) in the Occluded Task as predictors (see results in Table S2 in Supplementary Information).

**Table 2:** Regression models predicting facial attractiveness (*N* = 32)

Model	*y*	*x*	*β*	*t*	*p*	*VIF*	*R*^2^	Adjusted *R*^2^	*F*
*2019*									
Model 1	Predicted	Sunglasses	.866	9.494	< .001	1.000	.750	.742	*F* = 90.139, *p* < .001
Model 1	Complete	Sunglasses	.820	7.857	< .001	1.000	.673	.662	*F* = 61.372, *p* < .001
*2022*									
Model 1	Predicted	Sunglasses	.800	7.312	< .001	1.000	.641	.629	*F* = 53.472, *p* < .001
Model 2	Predicted	Sunglasses	.576	4.899	< .001	1.523	.737	.719	*F* = 40.620, *p* < .001
		Mask	.383	3.259	= .003	1.523			
Model 1	Complete	Sunglasses	.757	6.339	< .001	1.000	.573	.558	*F* = 40.182, *p* < .001
Model 2	Complete	Sunglasses	.480	3.949	< .001	1.523	.719	.700	*F* = 37.125, *p* < .001
		Mask	.473	3.890	= .001	1.523			

The 2019 results showed that only the attractiveness of sunglasses-occluded faces (i.e., the bottom half) significantly predicted attractiveness ratings for both predicted and complete faces. However, in 2022, the attractiveness of both sunglasses-occluded faces (bottom half) and mask-occluded faces (top half) significantly predicted attractiveness ratings for both predicted and complete faces. These findings suggest that the widespread experience with mask-wearing in 2022 may have increased the importance of the top half of the face in judging overall facial attractiveness or have enhanced their ability to infer missing features and perceive beauty even with partial facial cues. This aligns with post-COVID-19 studies (e.g., Gao et al., 2025; Liu et al., 2022) that presented participants with only top or bottom halves of faces. The prevalent use of masks during this period likely increased individuals’ exposure to and familiarity with perceiving faces with limited visual information, potentially increasing the importance of the top half of the face in judging overall facial attractiveness or enhancing their ability to infer missing features and perceive beauty even with partial facial cues.

## Experiment 2: Exploring “Filling in” strategies post-COVID-19

Experiment 2 explored the cognitive processes underlying the “Less is More” effect by examining how participants mentally “filled in” missing facial information. We investigated three hypotheses: the Most Beautiful Filling hypothesis (Sadr & Krowicki, 2019), which suggests individuals imagine the most attractive completion, and the Average/Typical Filling hypothesis (Kramer & Jones, 2022; Orghian & Hidalgo, 2020), which posits the use of an average or typical facial representation, and the Moderate Attractiveness Filling hypothesis (Kamatani et al., 2023), which posits that people use a representation of moderate attractiveness, rather than an average one, to infer own-race occluded faces. To test these hypotheses, participants viewed faces occluded by sunglasses and selected the most likely face from a high-attractiveness, average, and low-attractiveness face. We selected sunglasses over masks because eyes generally receive higher attractiveness ratings (Liu et al., 2022; Saegusa & Watanabe, 2016). This was also supported by our Experiment 1, where faces with visible eyes (mask condition, *M* = 5.397, SD = 0.098) were rated more attractive than faces with only lower face visible (sunglasses condition, *M* = 4.939, SD = 0.105). Thus, sunglasses occlusion provided a more conservative baseline for assessing “filling in” strategies. Data for Experiment 2 were collected in 2021.

## Method

### Participants

Prior power analysis was conducted using PANGEA (Westfall, 2016). A sample size of 15 was required to reveal a medium effect (*d* = 0.45) of 2 (Inherent attractiveness) × 3 (Test face) with power of 0.813 and Replicates = 20, var (error) = 0.333, var (Participants × Inherent attractiveness × Test face) = 0.083. To align with the previous sub-experiment in Experiment 1 and boost statistical power, we used a comparable sample size in the present experiment.

Thirty-two undergraduate students (21 females, 11 males; *M*_age_ = 20.66 years, SD_age_ = 1.69 years) from XXX University participated in Experiment 2. All provided informed consent before participating and were paid after the experiment. All had normal or corrected-to-normal vision and were unfamiliar with the faces used in the experiment.

### Stimuli

In Experiment 2, we used forty original faces [20 male (10 high-attractiveness, 10 low-attractiveness) and 20 female (10 high-attractiveness, 10 low-attractiveness)] from Experiment 1 as stimuli.

**Fig. 5:**
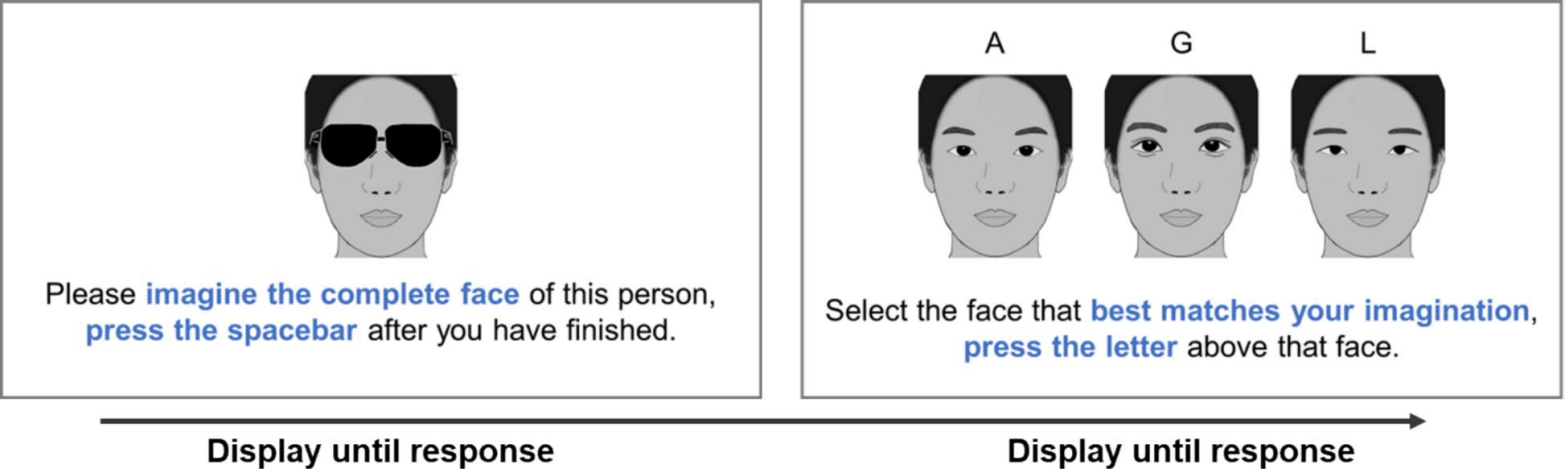
The procedure illustration of experiment 2. *Note.* Due to copyright restrictions, caricatures are used for illustrative purposes here. The actual experiment utilized real facial photographs. The face with sunglasses was always shared the same lower half as those three test faces. In the provided example, the left test face represents the face with average eyes, the middle test face represents the face with high-attractiveness eyes, and the right test face represents the face with low-attractiveness eyes

The forty original faces with sunglasses occlusion were used for the imagination phase (as depicted in the left panel of Fig. 5).

**High and Low-Attractiveness Test Faces**. To create the test faces, we composited 10 different sets of eyes from 10 high-attractiveness female faces with each original female face, yielding 10 high-attractiveness female test faces (actually 9 test faces + 1 self-composite face) per original female face. This process was repeated using 10 different sets of eyes from low-attractiveness female faces to generate 10 low-attractiveness female test faces for each original female face. The same procedure was then applied to for male faces. This resulted in 200 high-attractiveness female test faces, 200 low-attractiveness test faces, 200 high-attractiveness male test faces, and 200 low-attractiveness male test faces.

**Fig. 6:**
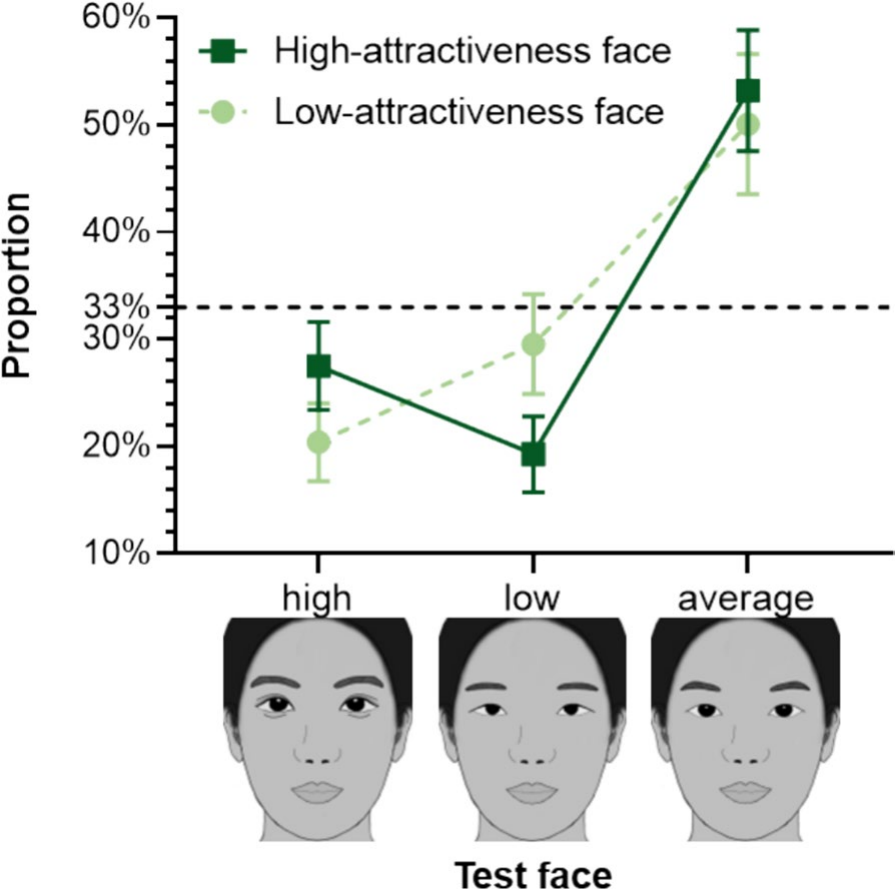
The Selection Rate of the Three Types of Test faces (high, low, average) for High-/Low-Attractiveness Faces. *Note*. The error bars represent the 95% confidence interval. The horizontal dashed line represents the random level (33%)

**Average Test Faces**. An average female face was generated by morphing the 20 original female faces. Similarly, an average male face was generated by morphing the 20 original male faces. Subsequently, the eye representations from the respective average faces (female or male) were replaced into each original face, creating 40 average test faces while maintaining gender consistency. Examples of the created test faces are shown in Fig. 6.

### Procedure

In each trial (see Fig. 5), a face occluded by sunglasses was displayed at the center of the screen. Participants were instructed to imagine its whole face and then press spacebar once they had finished the imagination.

Following this, three test faces (high-attractiveness, low-attractiveness, and average) were presented horizontally on the screen. The order of the test faces was randomized. Participants were then asked to select the test face that best matched their imagination by pressing the “A” key for the left face, the “G” key for the middle face, and the “L” key for the right face.

There were 40 trials in total, each presenting a different original face occluded by sunglasses, displayed in random order. For each occluded face, the high-attractiveness and the low-attractiveness test faces were randomly selected from a set of 9 pre-defined test faces that shared the same lower half as the occluded face.

Experiment 2 used E-Prime 2.0 for stimulus presentation and response recording. Stimuli were presented on a 23-inch 1920 × 1080 pixels CRT monitor with a 60 Hz refresh rate.

## Results and discussion

A 2 (Inherent Attractiveness: high, low) × 3 (Test Face: high, low, average) repeated-measures ANOVA for selection rates revealed a significant main effect of Test Face [*F*(1.341,41.586) = 35.319, *p* < .001, $\eta_{p}^{2} =$.533], but no significant main effect of Inherent Attractiveness [*F*(1,31) < 0.001, *p* = 1.000, $\eta_{p}^{2} <$.001]. Importantly, the interaction between Inherent attractiveness and Test face was significant [*F*(2,62) = 17.247, *p* < .001, $\eta_{p}^{2} =$.357] (see Fig. 6).

For occluded high-attractiveness faces, participants selected the average test face (*M* = 0.532, *SD* = 0.156) significantly more often than both the high-attractiveness test faces (*M* = 0.275, *SD* = 0.114) [*t*(31) = 5.683, *p*_bonf_ < .001, Cohen’s* d* = 1.930] and the low-attractiveness test face (*M* = 0.193, *SD* = 0.097) [*t*(31) = 8.182, *p*_bonf_ < .001, Cohen’s* d* = 2.546]. Participants selected the high-attractiveness test faces significantly more often than low-attractiveness test faces [*t*(31) = 3.245, *p*_bonf_ = .042, Cohen’s *d* = 0.616).

Similarly, for occluded low-attractiveness faces, the average test faces (*M* = 0.501, *SD* = 0.181) was selected significantly more often than the high-attractiveness test face (*M* = 0.204, *SD* = 0.099) [*t*(31) = 6.402, *p*_bonf_ < .001, Cohen’s* d* = 2.229] and the low-attractiveness test face (*M* = 0.295, *SD* = 0.129) [*t*(31) = 3.889, *p*_bonf_ < .001, Cohen’s* d* = 1.543]. Participants selected the low-attractiveness test faces significantly more often than high-attractiveness test faces [*t*(31) = 3.629, *p*_bonf_ = .015, Cohen’s *d* = 0.686).

These results, particularly the strong preference for faces with average eyes over those with high-attractiveness eyes, lend greater support to the Average/Typical Filling hypothesis (Kramer & Jones, 2022; Orghian & Hidalgo, 2020) than the Most Beautiful Filling hypothesis (Sadr & Krowicki, 2019). However, a crucial nuance arises when we consider the averageness effect in facial attractiveness. As research has shown, faces that are closer to the population average are often rated as more attractive than individual, non-morphed faces (Langlois & Roggman, 1990). Given this, it is highly plausible that the “average” eyes used in our study were cognitively perceived as the most attractive. This blurs the distinction between the two competing hypotheses. Our results could, in fact, be interpreted as evidence for the Most Beautiful Filling hypothesis (Sadr & Krowicki, 2019), if one perceives the “average” eyes as the “most beautiful”.

Furthermore, participants tended to select high-attractiveness (vs. low-attractiveness) completions for inherent high-attractiveness faces and low-attractiveness (vs. high-attractiveness) completions for inherent low-attractiveness faces. This pattern did not support Moderate Attractiveness Filling hypothesis (Kamatani et al., 2023). To explain this finding, we propose a novel Matching Filling hypothesis: individuals tend to mentally complete occluded faces with features that align with the perceived attractiveness of the visible regions. While our data suggest that this matching effect is a factor, it is outweighed by the general tendency to use average features. Therefore, we posit that the “matching” effect may only come into play when the brain is forced to choose between non-average options.

## Experiment 3: “Less is More” effect and filling-in strategies for familiar faces and unfamiliar faces

Following the demonstration of the “Less is More” effect for unfamiliar faces (Experiment 1) and the investigation of filling-in strategies for them (Experiment 2), Experiment 3 aimed to explore filling-in processes for familiar faces (Experiments 3a and 3b) and to determine if the “Less is More” effect extends to familiar faces (Experiment 3b).

Given ERP evidence suggesting the role of memory in filling missing facial information (Righart et al., 2011), we hypothesized that the underlying mechanisms for familiar faces would differ from those for unfamiliar faces, likely involving memory-based representations. We also examined the applicability of the Matching Filling hypothesis to both familiar and unfamiliar faces. To this end, Experiment 3 introduced a new category of test faces—self-composite test faces—in addition to the high-attractiveness, low-attractiveness, and average test faces used in Experiment 2. Data for Experiment 3 were collected in 2023.

## Method

### Participants

Prior power analysis was conducted using PANGEA (Westfall, 2016). A sample size of 8 was required in Experiment 3a to reveal a medium effect (*d* = 0.45) of 2 (Familiarity) × 2 (Inherent Attractiveness) × 4 (Test face) with power of 0.837 and Replicates = 10, var (error) = 0.2, var (Participants × Familiarity × Inherent Attractiveness × Test face) = 0.04.

And a sample size of 8 was required in Experiment 3b to reveal a medium effect (*d* = 0.45) of 2 (Familiarity) × 2 (Inherent Attractiveness) × 3 (Task) with power of 0.803 and Replicates = 10, var (error) = 0.2, var (Participants × Familiarity × Inherent Attractiveness × Task) = 0.04.

To align with the previous two experiments and boost statistical power, we employed a comparable sample size in each sub-experiment. A total of 70 undergraduate students from XXX University participated in Experiment 3: Thirty-four participants (16 females, 18 males; *M*_age_ = 21.94 years, SD_age_ = 1.35 years) in Experiment 3a and thirty-six participants (18 females, 18 males; *M*_age_ = 20.9 years, SD_age_ = 1.8 years) in Experiment 3b. All participants provided informed consent before participating and received compensation for their time after the experiment. All participants reported having normal or corrected-to-normal vision and were unfamiliar with the faces used in the experiment.

### Stimuli

Experiment 3 used the same 40 original faces and their sunglasses-occluded versions from Experiments 1 and 2. The 40 occluded faces were divided into two sets (A and B), each containing 10 female faces (5 high-attractiveness, 5 low-attractiveness) and 10 male faces (5 high-attractiveness, 5 low-attractiveness). Expanding on the test faces from Experiment 2 (high-attractiveness, low-attractiveness, and average), Experiment 3 introduced self-composite test faces. These were created by combining the eye region of each original face with its own original face. We used these self-composites, rather than the original full faces, to ensure the consistency in edge blurring across all test faces, avoiding the sharp edges of the originals that might make them easily identifiable. This design allowed us to investigate whether participants “filled in” the missing information for familiar faces based on their memory for those faces.

### Design

A 2 (Familiarity: familiar, unfamiliar) × 2 (Inherent attractiveness: high, low) × 4 (Test face: high, low, average, self) within-subject design was used in Experiment 3a and 3b.

Additionally, a 2 (Familiarity: familiar, unfamiliar) × 2 (Inherent attractiveness: high, low) × 3 (Task: Occluded, Predicted, Complete) within-subject design was used in Experiment 3b.

### Procedure

**Fig. 7:**
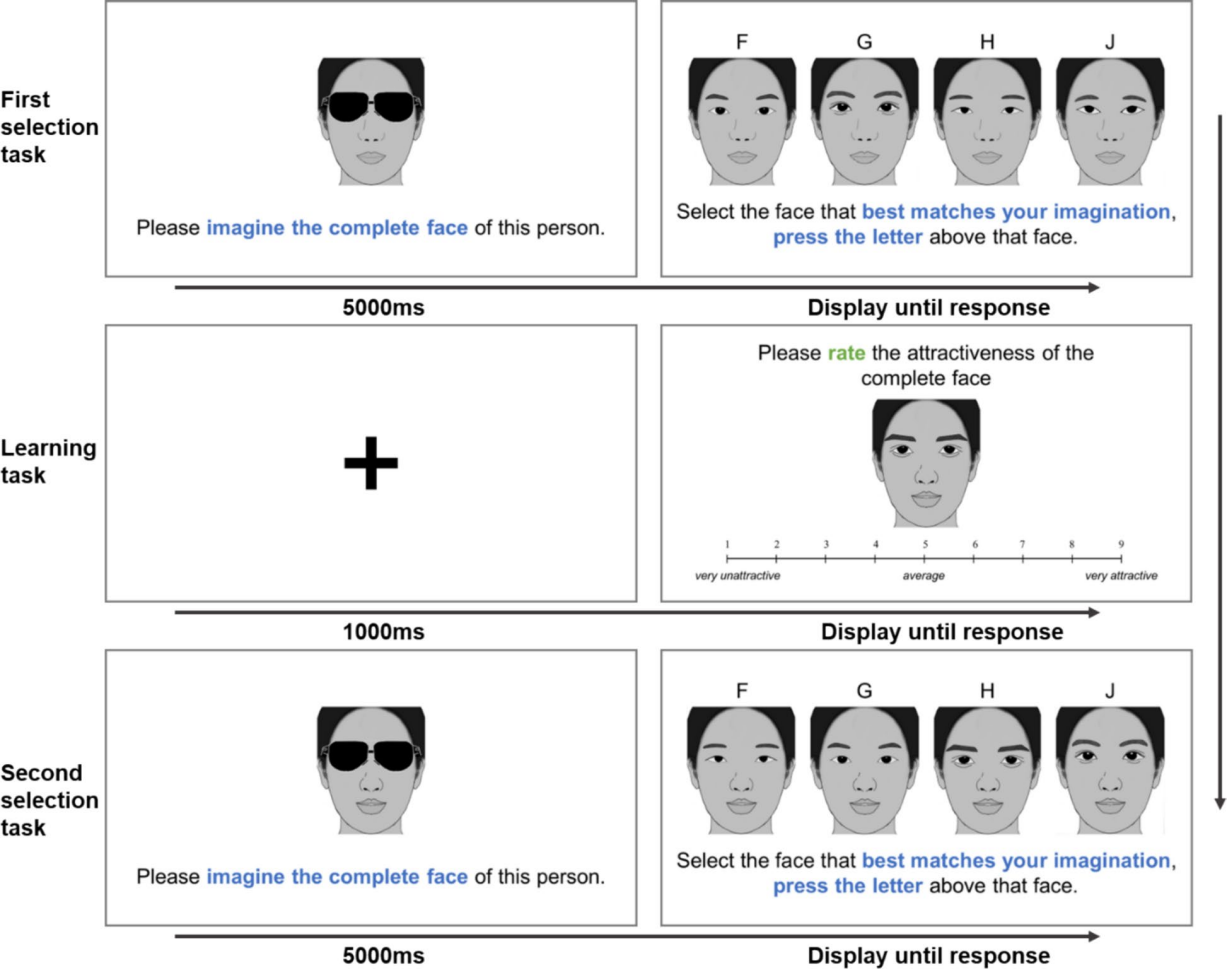
The procedure illustration of experiment 3a. *Note.* The faces presented in the First Selection Task (unfamiliar faces) and in the second selection task (familiar faces) were drawn from different sets. In this example, the four test faces presented horizontally, from left to right, in the First Selection Task: Average, high, low, self; In the second selection task: Low, average, self, and high

As illustrated in Fig. 7, Experiment 3a comprised three sequential blocks: first selection task (unfamiliar phase), learning task, and second selection task (familiar phase).

**First Selection Task.** The procedure of each trial was similar to that in Experiment 2, except that the faces with sunglasses would appear 5000 ms, and participants selected from four test faces instead of three. There were 20 trials for 20 faces (10 males, 10 females) with sunglasses from one set (A or B) in a random order.

**Learning Task.** Each trial began with a 1000 ms fixation cross. Following this, an original face (not previously presented in the First Selection Task) was displayed at the center of the screen. Participants were asked to remember the face image and to rate the attractiveness of the face on a 9-point scale below it (1 = *very unattractive,* 5 = *average,* 9 = *very attractive*). There were 20 trials for the 20 original faces from the other set (B or A) in a random order. This task served two purposes: to familiarize participants with the faces that would be used in the Second Selection Task, and to test whether the inherent attractiveness manipulation was effective and to be treated as Complete task in Experiment 3b.

**Second Selection Task.** The procedure was identical to the First Selection Task with one key difference: the faces presented in this block were the same as those presented in the Learning Task, while the faces in the First Selection Task were entirely different. This design aimed to examine whether the “filling-in” strategies differ for familiar versus unfamiliar faces.

To control for potential order effects, participants were randomly assigned to two halves: One half completed the First Selection Task with faces from Set A, followed by the Learning Task with Set B faces, and finally the Second Selection Task with Set B faces. The other half completed the First Selection Task with Set B faces, followed by the Learning Task with Set A faces, and finally the Second Selection Task with Set A faces. A 1-min rest period was provided between each block.

Experiment 3b followed a similar procedure as Experiment 3a, with a key difference: two additional rating tasks were included within both the First and Second Selection Task blocks. Before making their selections in each trial, participants were asked to rate the attractiveness of the faces occluded by sunglasses (i.e., the Occluded task) and predict the attractiveness of the complete face (i.e., the Predicted task). Following these two ratings, participants then proceeded to select the test face that best matched their mental “filling-in” of the missing facial information.

Experiment 3 was conducted using E-Prime 2.0 software on a computer with a 1920 × 1080 pixels resolution and a 60 Hz refresh rate.

## Results and discussion

### Different selection strategies for familiar and unfamiliar faces

To investigated whether participants employed different filling-in strategies for familiar and unfamiliar faces, we analyzed the selection rates of each test face types in Experiments 3a and 3b using a 2 (Experiment: 3a, 3b) × 2 (Familiarity: familiar, unfamiliar) × 2 (Inherent attractiveness: high, low) × 4 (Test face: high, low, average, self) mixed-design ANOVA. The results (see Table 3) showed no significant main effect of Experiment, indicating that the selection strategies used in Experiment 3a and 3b were similar.

**Table 3:** Results of 2 (Experiment) × 2 (Familiarity) × 2 (Inherent attractiveness) × 5 (Test Face) Mixed-design Repeated-measure ANOVA in Experiment 3

Effect	*F*	*df*	*p*	$\eta_{p}^{2}$
Experiment (E)	− 5.544 × 10^−15^	(1, 68)	1.000	− 8.153 × 10^−17^
Familiarity (F)	− 8.889 × 10^−14^	(1, 68)	1.000	− 1.307 × 10^−15^
Inherent attractiveness (A)	− 2.786 × 10^−13^	(1, 68)	1.000	− 4.098 × 10^−15^
Test face (T)	**104.817**	**(2.071, 140.859)**	** < .001**	**.607**
E × F	− 8.451 × 10^−14^	(1, 68)	1.000	− 1.243 × 10^−15^
E × A	− 5.853 × 10^−14^	(1, 68)	1.000	− 8.608 × 10^−16^
E × T	2.272	(2.071, 140.859)	.105	.032
F × A	^−^2.177 × 10^−15^	(1, 68)	1.000	^−^3.202 × 10^−17^
F × T	**22.063**	**(2.149, 146.156)**	** < .001**	**.245**
A × T	**5.832**	**(2.389, 162.480)**	**.002**	**.079**
E × F × A	^−^3.402 × 10^−17^	(1, 68)	1.000	^−^5.003 × 10^−19^
E × F × T	1.636	(2.149, 146.156)	.196	.023
E × A × T	1.963	(2.389, 162.480)	.135	.028
F × A × T	**3.601**	**(2.379, 161.790)**	**.023**	**.050**
E × F × A × T	2.024	(2.379, 161.790)	.126	.029

Bold indicates significant effects

**Fig. 8:**
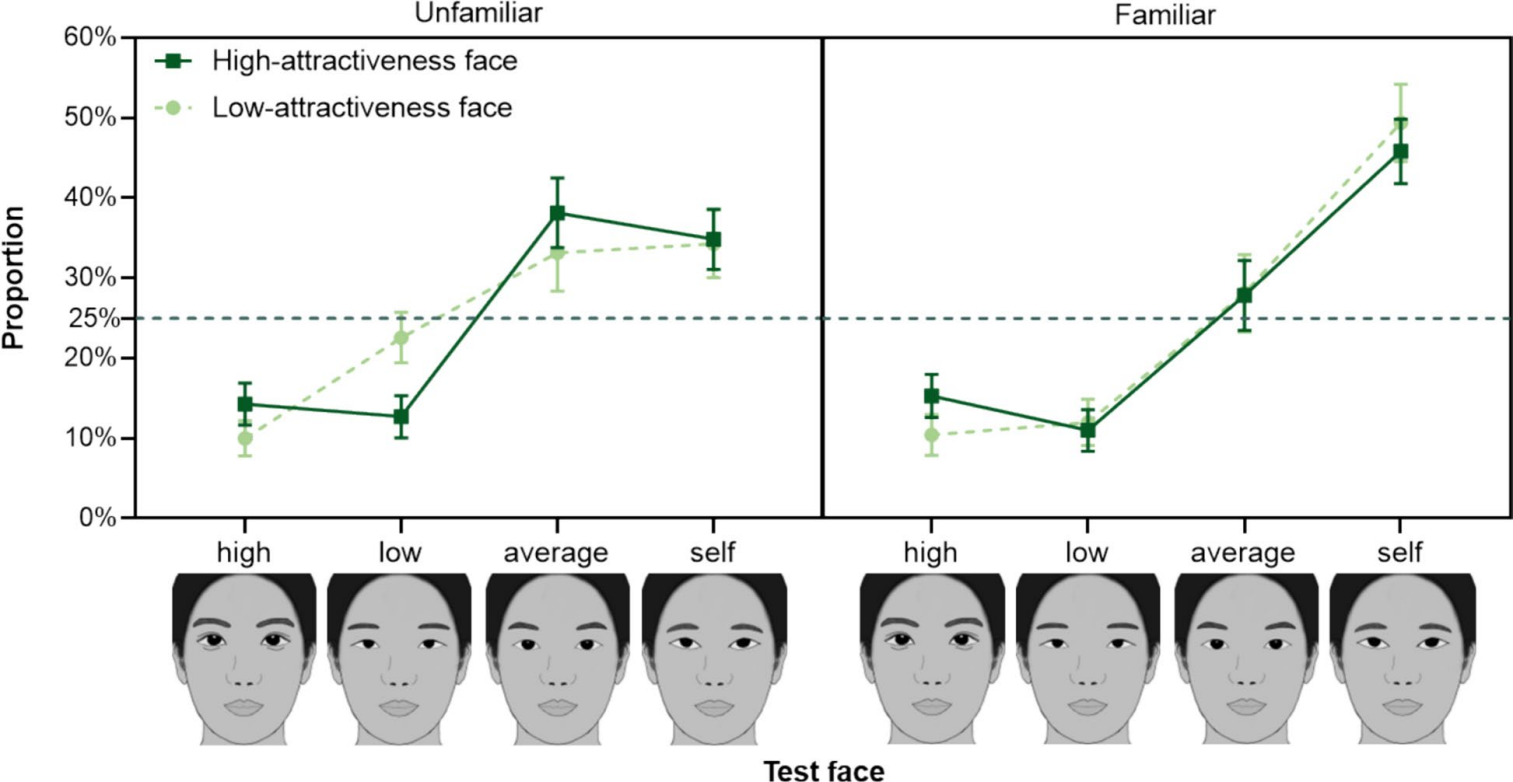
The Selection Rate as a Function of Test Face, Inherent attractiveness, and Familiarity in Experiment 3. *Note*. The error bars represent the 95% confidence interval. The horizontal dashed line represents the chance level (25%)

We observed a significant main effect of Test face. Importantly, this effect was further modulated by both Familiarity and Inherent attractiveness, as evidenced by a significant three-way interaction among these factors (see Fig. 8). We conducted separated 2 (Inherent attractiveness) × 4 (Test face) ANOVA for unfamiliar and familiar faces.

**Unfamiliar Faces.** The ANOVA for unfamiliar faces revealed no significant main effect of Inherent attractiveness [*F*(1, 69) = 2.750 × 10^−14^, *p* = 1.000, $\eta_{p}^{2} < .001$], but a significant main effect of Test face [*F*(2.107, 145.392) = 52.486, *p* < .001, $\eta_{p}^{2} = .432$] and a significant interaction between them [*F*(2.309, 159.329) = 7.432, *p* < .001, $\eta_{p}^{2} =$.097].

Simple effect tests of this interaction showed that for high-attractiveness occluded faces, the selection rates for average test faces (*M* = 0.381, SD = 0.182) and self-composite test faces (*M* = 0.349, SD = 0.158) did not significantly differ [*t*(69) = 1.124, *p*_bonf_ = 1.000, Cohen’s* d* = 0.219]. However, both were selected significantly more often than high-attractiveness test faces (*M* = 0.143, SD = 0.110) [average vs. high: *t*(69) = 8.160, *p*_bonf_ < .001, Cohen’s* d* = 1.593; self vs. high: *t*(69) = 7.036, *p*_bonf_ < .001, Cohen’s* d* = 1.373] and low-attractiveness test faces (*M* = 0.127, SD = 0.110) [average vs. low: *t*(69) = 8.697, *p*_bonf_ < .001, Cohen’s* d* = 1.698; self vs. low: *t*(69) = 7.573, *p*_bonf_ < .001, Cohen’s* d* = 1.478]. The selection rates of high-attractiveness and low-attractiveness test faces did not significantly differ [*t*(69) = 0.537, *p*_bonf_ = 1.000, Cohen’s* d* = 0.105].

For low-attractiveness occluded faces, the selection rates for average test faces (*M* = 0.331, SD = 0.200) and self-composite test faces (*M* = 0.343, SD = 0.177) did not significantly differ [*t*(69) = 0.391, *p*_bonf_ = 1.000, Cohen’s* d* = 0.076]. Both were selected significantly more often than low-attractiveness test faces (*M* = 0.226, SD = 0.132) [average vs. low: *t*(69) = 3.616, *p*_bonf_ = .009, Cohen’s* d* = 0.706; self vs. low: *t*(69) = 4.007, *p*_bonf_ = .002, Cohen’s* d* = 0.782]. Furthermore, low-attractiveness test faces were selected more often than high-attractiveness test faces (*M* = 0.100, SD = 0.093) [*t*(69) = 4.300, *p*_bonf_ < .001, Cohen’s* d* = 0.839].

Consistent with Experiment 2, participants preferred average test faces over high-attractiveness test faces for occluded unfamiliar faces, supporting the Average/Typical Filling hypothesis (Kramer & Jones, 2022; Orghian & Hidalgo, 2020) over the Most Beautiful Filling hypothesis (Sadr & Krowicki, 2019). Additionally, the tendency to select high-attractiveness completions for high-attractiveness faces and low-attractiveness completions for low-attractiveness faces for unfamiliar faces supports the Matching Filling hypothesis.

**Familiar faces.** The 2 (Inherent attractiveness) × 4 (Test face) ANOVA for familiar faces revealed no significant interaction between inherent attractiveness and test face [*F*(2.470, 170.464) = 2.018, *p* = .125, $\eta_{p}^{2} =$.028] and no main effect of inherent attractiveness [*F*(1, 69) = 1.009 × 10^−13^, *p* = 1.000, $\eta_{p}^{2} < .001$], but a significant of main effect of test face [*F*(2.023, 139.563) = 92.095, *p* < .001, $\eta_{p}^{2} =$.572]. Post hoc tests showed significantly higher selection rates for self-composite test faces (*M* = 0.476, *SD* = 0.152) compared to average test faces (*M* = 0.280, SD = 0.163) [*t*(69) = 5.673, *p*_bonf_ < .001, Cohen’s* d* = 1.260], indicating a strong influence of memory cues in filling in occluded familiar faces, consistent with the ERP evidence in Righart et al. (2011). Selection rates for average test faces were also significantly higher than that for high-attractiveness test face (*M* = 0.129, SD = 0.087) [*t*(69) = 5.958, *p*_bonf_ < .001, Cohen’s* d* = 0.972], consistent with previous findings and supporting the Average/Typical Filling hypothesis (Kramer & Jones, 2022; Orghian & Hidalgo, 2020) over the Most Beautiful Filling hypothesis (Sadr & Krowicki, 2019). No significant difference was found between the selection rates of high- and low-attractiveness test faces (*M* = 0.115, SD = 0.086) [*t*(69) = 0.943, *p*_bonf_ = 1.000, Cohen’s* d* = 0.087]. These results suggest that participants did not primarily employ a *matching-attractiveness* strategy for familiar faces. Instead, the higher selection rates for self-composite test faces compared to average test faces indicate that participants likely adopted a *matching-identity* strategy when mentally completing familiar faces.

### Effect of familiarity and inherent attractiveness on “Less is More”

To examine the “Less is More” effect for familiar faces, we analyzed attractiveness ratings from Experiment 3b using a 2 (Familiarity: familiar, unfamiliar) × 2 (Inherent attractiveness: high, low) × 3 (Task: Occluded, Predicted, Complete) repeated-measures ANOVA.

**Fig. 9:**
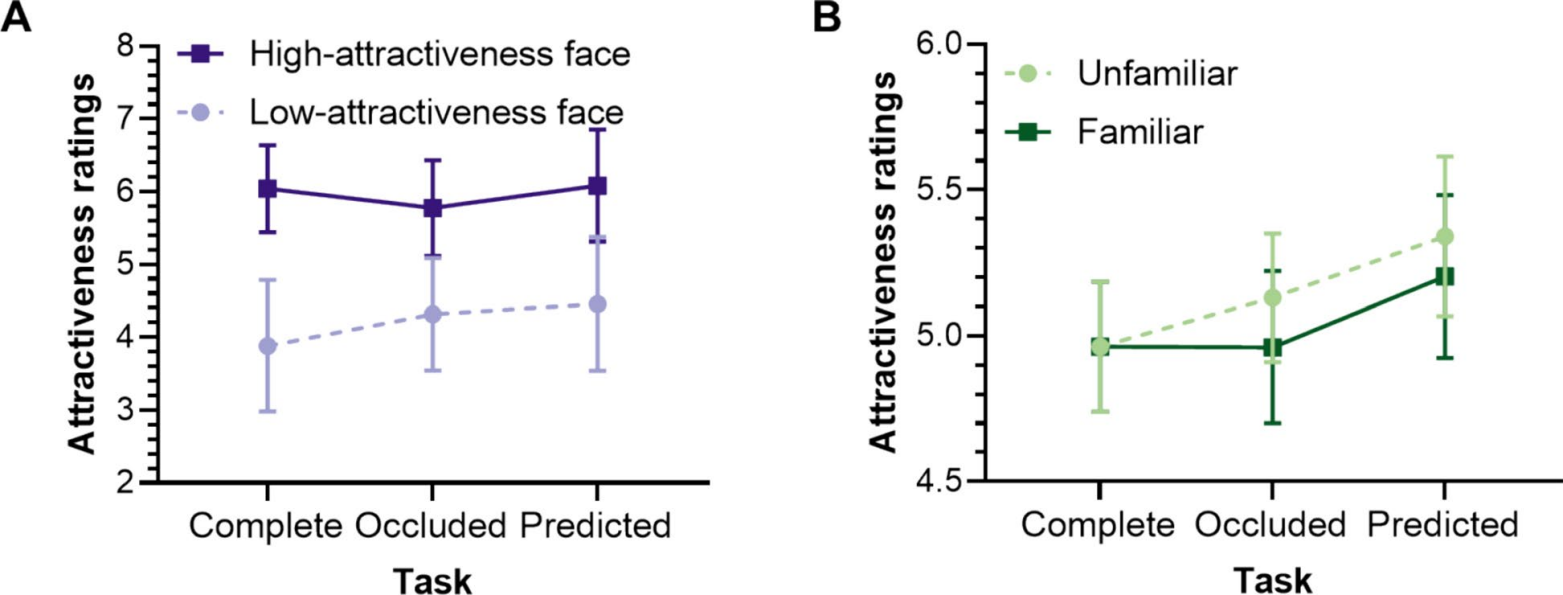
Attractiveness Ratings for High-/Low-Attractiveness Faces in Three Tasks in Experiment 3. *Note*. The error bars represent the 95% confidence interval

A significant main effect of Inherent attractiveness was observed [*F*(1,35) = 279.264, *p* < .001, $\eta_{p}^{2}$ = .889], with high-attractiveness faces (*M* = 5.966, SD = 0.605) rated significantly more attractive than low-attractiveness faces (*M* = 4.220, SD = 0.820). A significant main effect of Task was also found [*F*(1.650,57.739) = 9.677, *p* < .001, $\eta_{p}^{2}$ = .217]. Importantly, the effect of Task was significantly modulated by Inherent attractiveness [*F*(2,70) = 34.686, *p* < .001, $\eta_{p}^{2}$ = .498] (see Fig. 9A).

Simple effect analyses for low-attractiveness faces revealed significantly higher attractiveness ratings in both the Occluded Task (*M* = 4.317, SD = 0.770) [*t*(35) = 5.092, *p*_bonf_ < .001, Cohen’s* d* = 0.525] and the Predicted Task (*M* = 4.460, SD = 0.918) [*t*(35) = 6.778, *p*_bonf_ < .001, Cohen’s* d* = 0.698] compared to the Complete Task (*M* = 3.885, SD = 0.902), demonstrating a “Less is More” effect consistent with Experiment 1. However, for high-attractiveness faces, the Occluded Task (*M* = 5.775, SD = 0.659) resulted in significantly lower attractiveness ratings compared to the Complete Task (*M* = 6.040, *S*D = 0.597) [*t*(35) =  − 3.127, *p*_bonf_ = .034, Cohen’s* d* =  − 0.322]. No significant difference was found between the Predicted Task (*M* = 6.083, SD = 0.770) and the Complete Task (*p* = 1.000). The absence of a “Less is More” for high-attractiveness faces in this experiment, unlike Experiment 1, may be attributed to the involvement of familiar faces or the exclusive use of sunglasses-occluded faces.

Importantly, although the main effect of Familiarity [*F*(1,35) = 3.018, *p* = .091, $\eta_{p}^{2}$ = .079] and its interaction with Task [*F*(1.598,55.924) = 2.248, *p* = .125, $\eta_{p}^{2}$ = .060] did not reach statistical significance (using a *p* < .05 threshold), their moderate effect sizes warrant consideration.

Simple effect tests of the Familiarity × Task interaction revealed a “Less is More” effect for unfamiliar faces: attractiveness ratings in the Predicted task (*M* = 5.340, *SD* = 0.809) were significantly higher than in the Complete task (*M* = 4.963, SD = 0.660) [*t*(35) = 3.627, *p*_bonf_ = .013, Cohen’s* d* = 0.459]. However, attractiveness ratings in the Occluded task (*M* = 5.131, SD = 0.652) were not significantly higher than in the Complete task [*t*(35) = 1.952, *p*_bonf_ = .825, Cohen’s* d* = 0.204]. There was a tendency that the attractiveness rating for Predicted task was higher than Occluded task [*t*(35) = 2.943, *p*_bonf_ = .080, Cohen’s* d* = 0.255]. In contrast, no “Less is More” effect was observed for familiar faces [Complete task (*M* = 4.963, SD = 0.660); Predicted vs. Complete: *t*(35) = 2.721, *p*_bonf_ = .141, Cohen’s* d* = 0.292; Occluded versus Complete: *t*(35) = 0.016, *p*_bonf_ = 1.000, Cohen’s* d* = 0.002]. The difference between the Predicted (*M* = 5.203, SD = 0.824) and Occluded task (*M* = 4.961, SD = 0.771) for familiar faces reached significance [*t*(35) = 4.081, *p*_bonf_ = .003, Cohen’s* d* = 0.293].

This pattern suggests that the “Less is More” phenomenon diminishes or disappears when faces become familiar. Based on the findings of Experiment 3a, we interpret this as participants relying more on memory cues (their prior experience with the complete face) to “fill in” missing information rather than engaging in “average representation” imagination.

The interaction between Familiarity and Inherent attractiveness [*F*(1,35) = 0.168, *p* = .684, $\eta_{p}^{2}$ = .005] and the three-way interaction [*F*(1.948,68.188) = 0.162, *p* = .845, $\eta_{p}^{2}$ = .005] were not statistically significant.

## General discussion

This study investigated the influence of experience on the “Less is More” effect and its underlying mechanism, yielding three key findings. First, experience modulates the “Less is More” effect, as evidenced by two observations: the effect was presented both before and after the pandemic but was more pronounced post-pandemic (in Experiment 1), and it was observed for unfamiliar face but not for familiar faces (Experiment 3b). Second, the cognitive mechanisms underlying the “Less is More” effect are experience-dependent (Experiment 3a & 3b). For unfamiliar faces, our findings primarily support the Average/Typical Filling hypothesis, with some evidence also aligning with our novel Matching Filling hypothesis. In contrast, the completion of familiar faces relied more on memory cues. Third, the “Less is More” phenomenon was evident for both high- and low-attractiveness faces across all experiments (except when familiar faces were involved in Experiment 3), although it was consistently more pronounced for low-attractiveness faces.

### Role of experience on “Less is More”

Experience appears to influence the “Less is More” effect in two primary ways. First, the phenomenon was evident both before and after the pandemic, but its magnitude increased in post-pandemic (in Experiment 1), consistent with previous research (e.g., Kamatani et al., 2021). This temporal enhancement could be linked to increased exposure to sanitary-masked faces due to the widespread mask-wearing, as individuals who use masks more frequently tend to rate the attractiveness of masked faces higher (Dudarev et al., 2022a, 2022b). While our study did not specifically use sanitary masks, the stronger “Less is More” effect in 2022 across various occlusion types suggest a broader influence of visual experience with partial faces. Specifically, for high-attractiveness faces, only mask occlusion significantly increased attractiveness in 2019, whereas in 2022, both mask and lean hand occlusions did. For low-attractiveness faces, sunglasses, mask, and lean hand occlusions all significantly increased attractiveness in 2019; however, in 2022, all occluded faces were rated significantly higher than the complete face. This suggest that the heightened “Less is More” effect post-pandemic may not solely be attributed to a shift in the perception of masks from a sign of illness to one of social compliance (Nakayachi et al., 2020). Instead, it may reflect a more general impact of increased visual experience with occluded faces, aligning with the established link between mere exposure (Zajonc, 1968) and positive evaluations (Courtice et al., 2023; Dudarev et al., 2022a, 2022b).

Second, the influence of experience is further highlighted by the finding that the “Less is More” effect was more pronounced for unfamiliar face than for familiar faces (Experiment 3). This difference may stem from participants relying more on stored memory representations of complete familiar faces when processing occluded versions, potentially overriding the “filling-in” process that enhances attractiveness for unfamiliar faces.

However, the role of experience with own-race versus other-race faces remains unclear. Our study tested only own-race faces; other-race faces were not included. Moreover, although previous research (e.g., Dudarev et al., 2022a, 2022b; Kamatani et al., 2023) reported racial differences in the effect, subsequent re-analysis of the data (Hewer & Lewis, 2024) revealed that the racial differences were actually linked to the initial attractiveness of the faces rather than by race itself. Therefore, further research is needed to isolate and understand the pure influence of race on the “Less is More” phenomenon.

### The mechanism of “Less is More”

While the consistency of the “Less is More” mechanism before and after the pandemic remains unexplored, its dependence on facial familiarity is evident (in Experiment 3). For unfamiliar faces, participants predominantly favored the Average/Typical Filling hypothesis (Kramer & Jones, 2022; Orghian & Hidalgo, 2020) over the Most Beautiful Filling hypothesis (Sadr & Krowicki, 2019). Although some may argue that average faces are the most attractive (Baudouin & Tiberghien, 2004; Langlois & Roggman, 1990; Rhodes & Tremewan, 1996), and thus the Most Beautiful Filling hypothesis cannot be entirely ruled out, our findings lean more strongly toward the former. First, the notion that average faces are universally most attractive (Langlois & Roggman, 1990) lacks definitive support, as some studies (e.g., DeBruine et al., 2007) indicate that highly attractive faces surpass average faces in attractiveness. Furthermore, if participants in our study were primarily exploring a Most Beautiful Filling strategy, we would have expected a significantly higher selection rate for attractive test faces, which wasn’t the case; their selection was comparable to low-attractiveness test faces.

Our findings provided initial, qualified support for the novel Matching Filling hypothesis. When forced to choose between non-average options, participants showed a tendency to match completion attractiveness to the inherent level of the visible region: high-attractiveness completions were more likely for originally high-attractive faces, and low-attractiveness completions for originally low-attractive faces. This result suggests a preference for mentally completing occluded faces with features congruent with the perceived attractiveness of the visible regions, possibly reflecting an anchor effect (e.g., Goller et al., 2018). That is, participants anchor their “filling-in” on the visible parts of the occluded faces. A similar matching tendency was observed for familiar faces, where completions matched with memory representations were favored.

Our finding that high-attractiveness faces (especially unfamiliar faces) can also exhibit the “Less is More” effect did not support a general occlusion effect (e.g., Miyazaki & Kawahara, 2016), where objects increased the attractiveness of less attractive faces by concealing flaws and decreased the attractiveness of highly attractive faces by obscuring desirable features. However, in Experiment 3, occluded high-attractiveness faces were rated less attractive than complete ones, aligning with the occlusion effect described by Miyazaki and Kawahara (2016). Nevertheless, a crucial difference exists: Experiment 3 employed sunglasses as the occluder, whereas in Experiment 1, sunglasses did not produce a “Less is More” effect for high-attractiveness faces, either pre- or post-COVID-19. Therefore, the reduced attractiveness of sunglasses-occluded high-attractiveness faces in Experiment 3 is likely not solely due to a general occlusion effect, but possibly due to the introduction of familiar faces in that experiment.

Interestingly, both the Occluded and the Predicted tasks could trigger the “Less is More” effect, though it was more pronounced in the predicted task. The mechanism for the “Less is More” effect in the occluded task might be the occlusion effect itself, where unattractive features of less attractive faces are concealed by occlusion. In contrast, the mechanism behind the “Less is More” effect in the predicted task may involve “filling in” an average feature to form a predicted face.

These results collectively suggest a flexible cognitive strategy in processing occluded faces, where the specific filling-in mechanism may adapt based on factors like facial attractiveness, facial familiarity, and the nature of the task (direct perception of an occluded face vs. mental prediction).

### Implication of our findings

Our study revealed that various natural occlusions have differing effects on perceived beauty. Mask occlusion, which typically leaves the eyes visible, consistently enhanced attractiveness ratings. This finding aligns with previous research on mask-occluded faces (e.g., Liu et al., 2022; Pazhoohi & Kingstone, 2022). Interestingly, we also found that a lean hand, a common photography pose, effectively increase attractiveness. Vertical hand covering and sunglasses showed a lesser enhancing effect on attractiveness ratings compared to masks, consistent with the findings of Liu et al. (2022). Interestingly, while sunglasses (which occlude the eyes) did not alter the perceived attractiveness of high-attractiveness faces, they did increase the attractiveness of low-attractiveness faces. This might be because viewers imagined more attractive eyes behind the sunglasses.

### Limitation

A limitation of this study is its exclusive use of sunglasses occlusion to investigate the “Less is More” mechanism. Since the eyes are crucial for facial attractiveness, variations in the occluded regions and the extent of occlusion could influence this mechanism (Pazhoohi & Kingstone, 2022; Jeong et al., 2023). Future research should include faces with occlusions in other areas, such as the mouth region, to explore how facial attractiveness is processed with limited information.

Another limitation of this study is the failure to adequately investigate potential face gender or participant gender differences in the “Less is More” effect. To properly investigate gender differences in this effect, future studies must strictly match the attractiveness levels of male and female faces and recruited a larger, more balanced sample of participants across genders.

Furthermore, we found no main effect or interaction effect of familiarity in our experiment. This suggests our current manipulation of familiarity may not have been sensitive enough to capture its potential influence on facial attractiveness judgments. Future research could refine familiarity manipulation by varying repetition frequency in the face learning phase to create a more nuanced gradient of familiarity. Exploring other dimensions of familiarity, such as emotional or semantic familiarity, would also provide a more comprehensive understanding of its role in facial attractiveness evaluations.

## Conclusion

This study investigated how experience influences the “Less is More” effect and its underlying mechanism, yielding three key findings. First, experience modulates the “Less is More” effect. The effect was presented both before and after the pandemic, but was more pronounced post-pandemic (Experiment 1). It was also observed for unfamiliar face but not for familiar faces (Experiment 3b). Second, the cognitive mechanisms underlying the “Less is More” effect are experience-dependent (Experiment 3a & 3b). For unfamiliar faces, our findings primarily support the Average/Typical Filling hypothesis, with some evidence also aligning with our novel Matching Filling hypothesis. In contrast, completing familiar faces relied more on memory cues. Third, the “Less is More” phenomenon was evident for both high- and low-attractiveness faces across our experiments (except when familiar faces were involved), although it was consistently more pronounced for low-attractiveness faces.

## Supplementary Information

Additional file1

## Data Availability

The data that support the findings of this study are available from the corresponding author upon reasonable request.
