# Supplementary material for: Unveiling the “Less is More” paradox: How experience and cognitive filling drive attractiveness in occluded faces
Source: Cogn Res Princ Implic. 2025 Nov 27;10:81. doi: 10.1186/s41235-025-00691-w (PMC12660576; doi:10.1186/s41235-025-00691-w)
Supplement: Supplementary file 1 — Additional file1 [file 41235_2025_691_MOESM1_ESM.docx]

**Supporting Information for**

Unveiling the “Less is More” Paradox: How Experience and Cognitive Filling Drive Attractiveness in Occluded Faces

Yurou Gao, Mengliang Cao, Ruoying Zheng, and Guomei Zhou*

Department of Psychology, Sun Yat-sen University

*Corresponding author – Guomei Zhou

**Email**: zhougm@mail.sysu.edu.cn

**This file includes:**

Tables S1 – S8

**Table S1**

*Simple Effect Analysis Results of the Interaction of Year × Inherent attractiveness × Occlusion in Experiment 1*

| Occlusion | *M* | *SD* | 95% CI | *M* difference | *SE* |
| --- | --- | --- | --- | --- | --- |
| **2019, high-attractiveness faces** | | | | | |
| complete | 5.602 | 0.129 | [5.345, 5.858] |  |  |
| sunglasses | 5.694 | 0.129 | [5.435, 5.952] | 0.092 | 0.094 |
| Mask | 6.155 | 0.129 | [5.897, 6.414] | 0.554*** | 0.095 |
| vertical hand | 5.674 | 0.139 | [5.397, 5.952] | 0.073 | 0.090 |
| lean hand | 5.772 | 0.138 | [5.495, 6.049] | 0.170 | 0.098 |
| **2019, low-attractiveness faces** | | | | | |
| complete | 3.641 | 0.160 | [3.321, 3.960] |  |  |
| sunglasses | 3.931 | 0.168 | [3.595, 4.267] | 0.291* | 0.087 |
| Mask | 4.205 | 0.159 | [3.888, 4.523] | 0.565*** | 0.091 |
| vertical hand | 3.687 | 0.161 | [3.365, 4.008] | 0.046 | 0.065 |
| lean hand | 4.005 | 0.156 | [3.694, 4.317] | 0.365** | 0.086 |
| **2022, high attractive faces** | | | | | |
| complete | 5.938 | 0.129 | [5.681, 6.194] |  |  |
| sunglasses | 5.791 | 0.129 | [5.533, 6.050] | -0.146 | 0.094 |
| mask | 6.616 | 0.129 | [6.358, 6.875] | 0.679*** | 0.095 |
| vertical hand | 6.151 | 0.139 | [5.873, 6.428] | 0.213 | 0.090 |
| lean hand | 6.285 | 0.138 | [6.008, 6.562] | 0.348** | 0.098 |
| **2022, low attractive faces** | | | | | |
| complete | 3.969 | 0.160 | [3.649, 4.288] |  |  |
| sunglasses | 4.417 | 0.168 | [4.081, 4.753] | 0.448*** | 0.087 |
| mask | 4.792 | 0.159 | [4.475, 5.110] | 0.823*** | 0.091 |
| vertical hand | 4.277 | 0.161 | [3.955, 4.598] | 0.308*** | 0.065 |
| lean hand | 4.662 | 0.156 | [4.350, 4.974] | 0.693*** | 0.086 |

*Note*. *M* difference refers to the difference compared to complete condition. *Stands for significant difference at .05, ** at .01, *** at .001.

**Table S2**

*Results of the Multiple Linear Regression of the Attractiveness in the Occluded Task on that in the Prediction Task and the Complete Task in Experiment 1 (N=32)*

| Model | x | *β* | t | *p* | *VIF* | *R^2^* | *Adjusted R^2^* | *F* |
| --- | --- | --- | --- | --- | --- | --- | --- | --- |
| y = Predicted | | | | | | | | |
| Model 1 | Sunglasses | .831 | 11.764 | < .001 | 1.000 | .691 | .686 | *F* = 138.393, *p* < .001 |
| Model 2 | Sunglasses | .498 | 5.583 | < .001 | 2.222 | .781 | .774 | *F* = 108.887, *p* < .001 |
|  | Vertical hand | .449 | 5.025 | < .001 | 2.222 |  |  |  |
| Model 3 | Sunglasses | .345 | 3.748 | < .001 | 2.824 | .820 | .811 | *F* = 91.386,  *p* < .001 |
|  | Vertical hand | .378 | 4.514 | < .001 | 2.347 |  |  |  |
|  | Mask | .290 | 3.622 | = .001 | 2.137 |  |  |  |
| y = Complete | | | | | | | | |
| Model 1 | Vertical hand | .823 | 11.399 | < .001 | 1.000 | .677 | .672 | *F* = 129.946, *p* < .001 |
| Model 2 | Vertical hand | .573 | 7.177 | < .001 | 1.681 | .769 | .761 | *F* = 101.372, *p* < .001 |
|  | Mask | .393 | 4.918 | < .001 | 1.681 |  |  |  |
| Model 3 | Vertical hand | .450 | 4.984 | < .001 | 2.347 | .791 | .781 | *F* = 75.808,  *p* < .001 |
|  | Mask | .291 | 3.378 | = .001 | 2.137 |  |  |  |
|  | Sunglasses | .252 | 2.545 | =.014 | 2.824 |  |  |  |

As shown in Table S2, when the vertical and lean hand conditions were included in our model, we found that the vertical hand occlusion significantly predicted both dependent variables (Predicted and Complete tasks), exhibiting a particularly strong effect on the Complete task.

The vertical hand condition demonstrated greater predictive power for complete faces than both the sunglasses and mask conditions. This difference likely stems from the amount of visible information. The vertical hand condition exposes approximately half of the face (the left or the right side), offering substantially more information about the full face compared to sunglasses (which expose only the nose and mouth) or masks (which primarily expose the eyes). This increased visual information may reduce the cognitive effort required for participants to “fill in” the missing parts, leading to more accurate predictions.

Interestingly, the lean hand condition failed to significantly predict either dependent variable, despite covering only one more eye than the mask condition, which was predictive. This disparity highlights the specific importance of key facial features. Th mask condition exposes both eyes, thereby providing a critical cue for holistic processing—the interocular distance. According to the traditional Chinese aesthetic principle of "Three Forehead and Five Eyes" (Lin & Zhou, 2021), interocular distance significantly influences judgments of facial attractiveness. This vital cue is largely absent in the lean hand condition, which exposes only one eye.

To investigate potential gender difference, we re-analyzed the data from Experiment 1, incorporating both face gender and participant gender as additional independent variables. Table S3 presents the statistical results related with the two variables, in comparison to the primary model presented in Table 1.

**Table S3**

*Additional Results of 2 (Year) × 2 (Inherent Attractiveness) × 2 (Task) × 5 (Occlusion) × 2 (Face Gender) × 2 (Participant Gender) Mixed-design ANOVA Versus Table 1 in Experiment 1*

| Effect | *F* | *df* | *p* | $\eta_{p}^{2}$ |
| --- | --- | --- | --- | --- |
| **Face Gender (FG)** | **31.993** | **(1,60)** | **< .001** | **.347** |
| Participant Gender (PG) | 0.442 | (1,60) | .509 | .007 |
| Y × FG | 0.073 | (1,60) | .787 | .001 |
| Y × PG | 1.079 | (1,60) | .303 | .018 |
| **A × FG** | **45.934** | **(1,60)** | **< .001** | **.434** |
| A × PG | 0.248 | (1,60) | .621 | .004 |
| T × FG | 3.625 | (1,60) | .062 | .057 |
| T × PG | 0.486 | (1,60) | .488 | .008 |
| **O × FG** | **9.968** | **(3.517,211.018)** | **< .001** | **.142** |
| O × PG | 1.198 | (3.497,209.792) | .313 | .020 |
| FG × PG | 2.592 | (1,60) | .113 | .041 |
| Y × A × FG | 0.849 | (1,60) | .361 | .014 |
| Y × A × PG | 0.566 | (1,60) | .455 | .009 |
| Y × T × FG | .017 | (1,60) | .896 | .000 |
| Y × T × PG | 0.690 | (1,60) | .410 | .011 |
| Y × O × FG | 0.834 | (3.517,211.018) | .492 | .014 |
| Y × O × PG | 0.296 | (3.497,209.792) | .857 | .005 |
| A × T × FG | 0.061 | (1,60) | .806 | .001 |
| A × T × PG | 1.867 | (1,60) | .177 | .030 |
| **A × O × FG** | **5.470** | **(3.441,206.489)** | **.001** | **.084** |
| A × O × PG | 1.131 | (3.372,202.303) | .340 | .019 |
| T × O × FG | 0.682 | (3.473,208.409) | .585 | .011 |
| T × O × PG | 2.140 | (3.333,199.952) | .090 | .034 |
| Y × FG × PG | 3.054 | (1,60) | .086 | .048 |
| A × FG × PG | 1.963 | (1,60) | .166 | .032 |
| T × FG × PG | 0.060 | (1,60) | .808 | .001 |
| **O × FG × PG** | **3.199** | **(3.517,211.018)** | **.018** | **.051** |
| Y × A × T × FG | 0.602 | (1,60) | .441 | .010 |
| Y × A × T × PG | 0.026 | (1,60) | .872 | .000 |
| Y × A × O × FG | 0.424 | (3.441,206.489) | .763 | .007 |
| Y × A × O × PG | 2.129 | (3.372,202.303) | .090 | .034 |
| **Y × T × O × FG** | **3.180** | **(3.473,208.409)** | **.019** | **.050** |
| Y × T × O × PG | 1.023 | (3.333,199.952) | .389 | .017 |
| A × T × O × FG | 1.206 | (3.218,193.097) | .309 | .020 |
| A × T × O × PG | 0.654 | (3.226,193.538) | .593 | .011 |
| Y × A × FG × PG | 0.370 | (1,60) | .546 | .006 |
| Y × T × FG × PG | 1.309 | (1,60) | .257 | .021 |
| Y × O × FG × PG | 1.991 | (3.517,211.018) | .106 | .032 |
| A × T × FG × PG | 1.122 | (1,60) | .294 | .018 |
| A × O × FG × PG | 1.001 | (3.441,206.489) | .401 | .016 |
| T × O × FG × PG | 0.086 | (3.473,208.409) | .979 | .001 |
| Y × A × T × O × FG | 0.584 | (3.218,193.097) | .638 | .010 |
| Y × A × T × O × PG | 1.964 | (3.226,193.538) | .116 | .032 |
| Y × A × T × FG × PG | 1.202 | (1,60) | .277 | .020 |
| Y × A × O × FG × PG | 1.573 | (3.441,206.489) | .191 | .026 |
| Y × T × O × FG × PG | 1.367 | (3.473,208.409) | .251 | .022 |
| A × T × O × FG × PG | 0.950 | (3.218,193.097) | .422 | .016 |
| Y × A × T × O × FG × PG | 0.388 | (3.218,193.097) | .776 | .006 |

*Note*. Bold indicates significant effects. Y indicates Year. A indicates Inherent Attractiveness. T indicates Task. O indicates Occlusion.

The main effect of Face Gender indicated that female faces (*M* = 5.272, *SD* = 0.088) were rated as significantly more attractive than male faces (*M* = 4.855, *SD* = 0.099). This finding immediately suggested that any observed gender effects might be confounded with facial attractiveness.

To specifically examine this potential confound, we analyzed participants’ attractiveness ratings for the complete female and male faces. The analysis revealed a a significant interaction between Face Gender and Inherent Attractiveness [*F* (1,63) = 14.609, *p* < .001, $\text{η}_{\text{p}}^{\text{2}}$ = .188]. Simple effects tests confirmed that: For high-attractiveness faces, female faces were rated higher than male faces [*M*_difference_ = 0.402, *SE* = 0.106, *p*_bonf_ < .001], while for low-attractiveness faces, the difference did not reach significant [*M*_difference_ = 0.044, *SE* = 0.089, *p*_bonf_ = .624]. This result indicated that the gender effects are confounded with attractiveness, especially for high-attractiveness faces. Since Attractiveness is known to modulate the effect of occlusion, and both Year and Attractiveness can influence the occlusion effect (see Table 1), it is difficult to isolate whether the observed effects concerning face gender are driven by face gender per se or by the higher attractiveness of the female faces. Therefore, the subsequent findings related to face gender should be interpreted with caution. Future studies designed to properly investigate the role of gender in the “Less is More” effect must strictly match the attractiveness levels of male and female stimuli.

The Face Gender effect was further modulated by both Inherent attractiveness and Occlusion, as evidenced by a significant three-way interaction among these factors. To decompose this higher-order interaction, we conducted separate 2 (Inherent attractiveness) × 5 (Occlusion) ANOVAs for female faces and male faces. The results of these simple effect analyses are presented in detail in Table S4.

**Table S4**

*Simple Effect Analysis Results of the Interaction of Inherent Attractiveness × Occlusion × Face Gender in Experiment 1*

| Occlusion | *M* | *SD* | 95% CI | *M* difference | *SE* |
| --- | --- | --- | --- | --- | --- |
| **Female, high-attractiveness faces** | | | | | |
| complete | 5.970 | 0.105 | [5.760, 6.181] |  |  |
| sunglasses | 6.060 | 0.093 | [5.874, 6.246] | 0.090 | 0.075 |
| mask | 6.824 | 0.095 | [6.633, 7.015] | 0.854*** | 0.073 |
| vertical hand | 6.150 | 0.111 | [5.928, 6.372] | 0.180 | 0.076 |
| lean hand | 6.435 | 0.107 | [6.221, 6.649] | 0.465*** | 0.073 |
| **Female, low-attractiveness faces** | | | | | |
| complete | 3.827 | 0.117 | [3.592, 4.061] |  |  |
| sunglasses | 4.266 | 0.112 | [4.043, 4.490] | 0.440*** | 0.076 |
| mask | 4.598 | 0.116 | [4.367, 4.830] | 0.772*** | 0.069 |
| vertical hand | 4.102 | 0.116 | [3.870, 4.334] | 0.276*** | 0.052 |
| lean hand | 4.485 | 0.117 | [4.252, 4.719] | 0.659*** | 0.075 |
| **Male, high attractive faces** | | | | | |
| complete | 5.569 | 0.105 | [5.359, 5.779] |  |  |
| sunglasses | 5.425 | 0.121 | [5.183, 5.667] | -0.144 | 0.087 |
| mask | 5.948 | 0.118 | [5.711, 6.184] | 0.379*** | 0.086 |
| vertical hand | 5.675 | 0.113 | [5.450, 5.900] | 0.106 | 0.069 |
| lean hand | 5.622 | 0.118 | [5.386, 5.858] | 0.053 | 0.087 |
| **Male, low attractive faces** | | | | | |
| complete | 3.783 | 0.125 | [3.533, 4.033] |  |  |
| sunglasses | 4.082 | 0.135 | [3.812, 4.352] | 0.299*** | 0.067 |
| mask | 4.399 | 0.126 | [4.147, 4.651] | 0.616*** | 0.075 |
| vertical hand | 3.861 | 0.124 | [3.614, 4.108] | 0.078 | 0.056 |
| lean hand | 4.182 | 0.121 | [3.941, 4.423] | 0.399*** | 0.065 |

*Note. M* difference refers to the difference compared to complete condition. *Stands for significant difference at .05, ** at .01, *** at .001.

We also observed a significant three-way interaction of Occlusion × Face Gender × Participant Gender. To further investigate this complex interaction, we conducted separate 2 (Participant Gender) × 5 (Occlusion) ANOVAs for female faces and male faces. The results of these simple effect analyses are presented in Table S5.

**Table S5**

*Simple Effect Analysis Results of the Interaction of Occlusion × Face Gender × Participant Gender in Experiment 1.*

| Occlusion | *M* | *SD* | 95% CI | *M* difference | *SE* |
| --- | --- | --- | --- | --- | --- |
| **Female faces, female participant** | | | | | |
| complete | 4.919 | 0.136 | [4.646, 5.191] |  |  |
| sunglasses | 5.232 | 0.130 | [4.972, 5.492] | 0.313* | 0.090 |
| mask | 5.645 | 0.128 | [5.389, 5.900] | 0.726*** | 0.087 |
| vertical hand | 5.130 | 0.142 | [4.846, 5.415] | 0.212 | 0.078 |
| lean hand | 5.447 | 0.139 | [5.169, 5.725] | 0.528*** | 0.090 |
| **Female faces, male participant** | | | | | |
| complete | 4.878 | 0.136 | [4.606, 5.151] |  |  |
| sunglasses | 5.095 | 0.130 | [4.835, 5.354] | 0.216 | 0.090 |
| mask | 5.778 | 0.128 | [5.523, 6.033] | 0.900*** | 0.087 |
| vertical hand | 5.122 | 0.142 | [4.838, 5.406] | 0.244* | 0.078 |
| lean hand | 5.473 | 0.139 | [5.196, 5.751] | 0.595*** | 0.090 |
| **Male faces, female participant** | | | | | |
| complete | 4.498 | 0.146 | [4.207, 4.790] |  |  |
| sunglasses | 4.634 | 0.169 | [4.297, 4.972] | 0.136 | 0.094 |
| mask | 4.997 | 0.157 | [4.683, 5.311] | 0.498*** | 0.100 |
| vertical hand | 4.766 | 0.150 | [4.466, 5.065] | 0.267** | 0.073 |
| lean hand | 4.796 | 0.152 | [4.492, 5.100] | 0.298* | 0.092 |
| **Male faces, male participant** | | | | | |
| complete | 4.853 | 0.146 | [4.562, 5.145] |  |  |
| sunglasses | 4.873 | 0.169 | [4.535, 5.211] | 0.020 | 0.094 |
| mask | 5.350 | 0.157 | [5.036, 5.664] | 0.497*** | 0.100 |
| vertical hand | 4.770 | 0.150 | [4.471, 5.070] | -0.083 | 0.073 |
| lean hand | 5.008 | 0.152 | [4.704, 5.312] | 0.155 | 0.092 |

*Note*. *Stands for *p* value = < .05, ***p* value = < .01, ****p* value = < .001.

To decompose the four-way interaction involving Year, Task, Occlusion, and Face Gender, we conducted separate 2 (Year) × 2(Task) × 5 (Occlusion) ANOVAs for female and male faces.

For female faces, the analysis yielded significant main effects for Occlusion [*F* (3.433, 212.848) = 47.889, *p* < .001, $\text{η}_{\text{p}}^{\text{2}}$ = .436] and Year [*F* (1,62) = 7.393, *p* = .008, $\text{η}_{\text{p}}^{\text{2}}$ = .107]. Crucially, a significant interaction of Occlusion × Year [*F* (3.433, 212.848) = 3.022, *p* = .025, $\text{η}_{\text{p}}^{\text{2}}$ = .046] was found. All other effects and interactions did not reach significance (*ps* > .119).

The interaction of Occlusion × Year for female faces was further examined by comparing occluded faces against complete faces. In 2019, the “Less is More” effect was observed for sunglasses [*M*_difference_ = 0.332, *SE* = 0.089, *p*_bonf_ = .004], mask [*M*_difference_ = 0.727, *SE* = 0.087, *p*_bonf_ < .001], and lean hand [*M*_difference_ = 0.433, *SE* = 0.089, *p*_bonf_ < .001], as all were rated significantly higher than complete faces (*M* = 4.709, *SD* = 0.136). In 2022, the effect persisted for the mask [*M*_difference_ = 0.899, *SE* = 0.087, *p*_bonf_ < .001], vertical hand [*M*_difference_ = 0.317, *SE* = 0.077, *p*_bonf_ = .001], and lean hand [*M*_difference_ = 0.691, *SE* = 0.089, *p*_bonf_ < .001], as they were rated higher than complete faces (*M* = 5.087, *SD* = 0.136).

However, for male faces, the results showed significant main effects of Year [*F* (1,62) = 4.780, *p* = .033, $\text{η}_{\text{p}}^{\text{2}}$ = .072], Task [*F* (1,62) = 8.659, *p* = .005, $\text{η}_{\text{p}}^{\text{2}}$ = .123], and Occlusion [*F* (3.556, 220.485) = 14.537, *p* < .001, $\text{η}_{\text{p}}^{\text{2}}$ = .190]. Critically, none of the interactions (including the Occlusion × Year interaction) reached significance (*ps* > .089).

To more directly test whether the change in the “Less is More” effect between 2019 and 2022 was dependent on gender or inherent attractiveness, we added face gender and participant gender as independent variables to 2 (Year: 2019, 2022) × 2 (Inherent attractiveness: high, low) × 3 (Task: Occluded, Predict, Complete) mixed-design ANOVA in Experiment 1. Table S6 provides the statistical results of all additional main effects and interaction effects resulting from the inclusion of these two variables.

**Table S6**

*Additional Results of 2 (Year) × 2 (Inherent Attractiveness) × 3 (Task) × 2 (Face Gender) × 2 (Participant Gender) Mixed-design ANOVA in Experiment 1*

| Effect | *F* | *df* | *p* | $\eta_{p}^{2}$ |
| --- | --- | --- | --- | --- |
| **Face Gender (FG)** | **26.771** | **(1,60)** | **<. 001** | **.309** |
| Participant Gender (PG) | 0.498 | (1,60) | .483 | .008 |
| Y × FG | 0.107 | (1,60) | .745 | .002 |
| Y × PG | 1.097 | (1,60) | .299 | .018 |
| **A × FG** | **40.894** | **(1,60)** | **<. 001** | **.405** |
| A × PG | 0.131 | (1,60) | .719 | .002 |
| **T × FG** | **18.653** | **(2,120)** | **<. 001** | **.237** |
| T × PG | 0.501 | (2,120) | .607 | .008 |
| FG × PG | 3.150 | (1,60) | .081 | .050 |
| Y × A × FG | 1.161 | (1,60) | .286 | .019 |
| Y × A × PG | 0.314 | (1,60) | .577 | .005 |
| Y × T × FG | 0.314 | (2,120) | .731 | .005 |
| Y × T × PG | 0.352 | (2,120) | .704 | .006 |
| A × T × FG | 1.523 | (2,120) | .222 | .025 |
| A × T × PG | 2.560 | (1.768,106.079) | .089 | .041 |
| Y × FG × PG | 2.621 | (1,60) | .111 | .042 |
| A × FG × PG | 2.458 | (1,60) | .122 | .039 |
| T × FG × PG | 2.821 | (2,120) | .063 | .045 |
| Y × A × T × FG | 1.231 | (2,120) | .296 | .020 |
| **Y × A × T × PG** | **3.868** | **(1.768,106.079)** | **.029** | **.061** |
| Y × A × FG × PG | 0.118 | (1,60) | .732 | .002 |
| Y × T × FG × PG | 1.772 | (2,120) | .174 | .029 |
| A × T × FG × PG | 1.694 | (2,120) | .188 | .027 |
| Y × A × T × FG × PG | 2.417 | (2,120) | .094 | .039 |

*Note*. Bold indicates significant effects. Y indicates Year. A indicates Inherent Attractiveness. T indicates Task.

We observed a significant main effect of Face Gender, which was modulated by Inherent attractiveness and Task, respectively. The interaction of Face Gender × Inherent attractiveness revealed that the gender difference in attractiveness ratings persisted across both inherent attractiveness levels. For high-attractiveness faces, female faces (*M* = 6.235, *SD* = 0.091) were rated significantly higher than male faces (*M* = 5.635, *SD* = 0.100; *M*_difference_ = 0.601, *SE* = 0.092, *p*_bonf_ < .001). For low-attractiveness faces, female faces (*M* = 4.184, *SD* = 0.107) were also rated significantly higher than male faces (*M* = 4.015, *SD* = 0.118; *M*_difference_ = 0.169, *SE* = 0.069, *p*_bonf_ = .017).

The interaction of Face Gender × Task revealed different patterns of the “Less is More” effect. For female faces, attractiveness ratings in the Occluded (*M* = 5.338, *SD* = 0.094) and Predicted (*M* = 5.393, *SD* = 0.084) tasks were both significantly higher than in the Complete task (*M* = 4.898, *SD* = 0.096) [Occluded: *M*_difference_ = 0.439, *SE* = 0.051, *p*_bonf_ < .001; Predicted: *M*_difference_ = 0.494, *SE* = 0.051, *p*_bonf_ < .001]. The difference between the Occluded and Predicted tasks was not significant (*M*_difference_ = 0.055, *SE* = 0.048, *p*_bonf_ = .762). However, for male faces, ratings in the Predicted task (*M* = 4.969, *SD* = 0.102) were significantly higher than in the Occluded task (*M* = 4.829, *SD* = 0.105; *M*_difference_ = 0.140, *SE* = 0.048, *p*_bonf_ = .016). Furthermore, ratings in the Occluded task were significantly higher than in the Complete task (*M* = 4.676, *SD* = 0.103; *M*_difference_ = 0.153, *SE* = 0.052, *p*_bonf_ = .013).

To further decompose the four-way interaction involving Year × Inherent attractiveness × Task × Participant Gender, we conducted separate 2 (Year) × 2 (Inherent attractiveness) × 3 (Task) ANOVAs for female and male participants. The results for both female and male participants showed significant main effects of Inherent attractiveness [female: *F* (1,30) = 178.983, *p* < .001, $\text{η}_{\text{p}}^{\text{2}}$ = .856; male: *F* (1,30) = 298.961, *p* < .001, $\text{η}_{\text{p}}^{\text{2}}$ = .909] and Task [female: *F* (2,60) = 26.368, *p* < .001, $\text{η}_{\text{p}}^{\text{2}}$ = .468; male: *F* (2,60) = 19.637, *p* < .001, $\text{η}_{\text{p}}^{\text{2}}$ = .396], alongside a significant interactions between these two factors [female: *F* (2,60) = 3.717, *p* = .030, $\text{η}_{\text{p}}^{\text{2}}$ = .110; male: *F* (1.484,44.520) = 12.828, *p* < .001, $\text{η}_{\text{p}}^{\text{2}}$ = .300].

Furthermore, for female participants only, the main effect of Year [*F* (1,30) = 5.274, *p* = .029, $\text{η}_{\text{p}}^{\text{2}}$ = .150] and the three-way interaction of Year × Inherent attractiveness × Task [*F* (2,60) = 5.496, *p* = .006, $\text{η}_{\text{p}}^{\text{2}}$ = .155] was significant. All other effects were non-significant (*ps* > .197). Detailed simple effect results of the three-way interaction are provided in Table S7.

**Table S7**

*The Interaction of Year × Inherent attractiveness × Task for Female Participants in Experiment 1*

| Occlusion | *M* | *SD* | 95% CI | *M*_diff vs. Complete_ | *SE* _vs. Complete_ | *M*_diff vs. Occluded_ | *SE* _vs. Occluded_ |
| --- | --- | --- | --- | --- | --- | --- | --- |
| **2019, high-attractiveness faces** | | | | | | | |
| Complete | 5.400 | 0.176 | [5.041, 5.759] |  |  |  |  |
| Occluded | 5.646 | 0.199 | [5.239, 6.052] | 0.246 | 0.115 |  |  |
| Predicted | 5.874 | 0.165 | [5.538, 6.211] | 0.474*** | 0.106 | 0.229 | 0.098 |
| **2019, low-attractiveness faces** | | | | | | | |
| Complete | 3.497 | 0.259 | [2.967, 4.027] |  |  |  |  |
| Occluded | 3.715 | 0.246 | [3.212, 4.218] | 0.218 | 0.107 |  |  |
| Predicted | 3.773 | 0.257 | [3.247, 4.298] | 0.276** | 0.080 | 0.058 | 0.096 |
| **2022, high-attractiveness faces** | | | | | | | |
| Complete | 5.956 | 0.176 | [5.597, 6.316] |  |  |  |  |
| Occluded | 6.195 | 0.199 | [5.788, 6.601] | 0.239 | 0.115 |  |  |
| Predicted | 6.271 | 0.165 | [5.934, 6.607] | 0.314* | 0.106 | 0.076 | 0.098 |
| **2022, low-attractiveness faces** | | | | | | | |
| Complete | 3.981 | 0.259 | [3.451, 4.511] |  |  |  |  |
| Occluded | 4.633 | 0.246 | [4.130, 5.136] | 0.652*** | 0.107 |  |  |
| Predicted | 4.542 | 0.257 | [4.017, 5.068] | 0.561*** | 0.080 | -0.091 | 0.096 |

*Note*. *Stands for *p* value = < .05, ***p* value = < .01, ****p* value = < .001.

For male participants, the interaction of Inherent attractiveness × Task was decomposed by examining the simple effect of Task separately for high- and low-attractiveness faces. For high-attractiveness faces, ratings in the Predicted task (*M* = 6.117, *SD* = 0.118) were significantly higher than both the Occluded task (*M* = 5.961, *SD* = 0.119; *M*_difference_ = 0.155, *SE* = 0.059, *p*_bonf_ = .038) and the Complete task (*M* = 5.861, *SD* = 0.131; *M*_difference_ = 0.256, *SE* = 0.078, *p*_bonf_ = .008). The difference between Occluded and Complete tasks was not significant (*M*_difference_ = 0.100, *SE* = 0.069, *p*_bonf_ = .468). For low-attractiveness faces, ratings in the Occluded (*M* = 4.279, *SD* = 0.127; *M*_difference_ = 0.409, *SE* = 0.071, *p*_bonf_ < .001) and Predicted (*M* = 4.378, *SD* = 0.124; *M*_difference_ = 0.508, *SE* = 0.071, *p*_bonf_ < .001) tasks were both significantly higher than the Complete task (*M* = 3.870, *SD* = 0.135). The difference between Occluded and Predicted tasks was not significant (*M*_difference_ = 0.099, *SE* = 0.071, *p*_bonf_ = .518).

**Table S8**

*The Interaction of Familiarity × Inherent attractiveness × Test Face in Experiment 3*

| Test face | M | SD | *Student’s t* | *M* difference | Cohen’s d |
| --- | --- | --- | --- | --- | --- |
| **Unfamiliar, high-attractiveness faces** | | | | | |
| High | 0.143 | 0.110 | -8.166 | -0.107*** | -0.976 |
| Low | 0.127 | 0.110 | -9.325 | -0.123*** | -1.115 |
| Average | 0.381 | 0.182 | 6.041 | 0.131*** | 0.722 |
| Self | 0.349 | 0.158 | 5.231 | 0.099*** | 0.625 |
| **Unfamiliar, low-attractiveness faces** | | | | | |
| High | 0.100 | 0.093 | -13.458 | -0.150*** | -1.609 |
| Low | 0.226 | 0.132 | -1.545 | -0.024 | -0.185 |
| Average | 0.331 | 0.200 | 3.400 | 0.081** | 0.406 |
| Self | 0.343 | 0.177 | 4.400 | 0.093*** | 0.526 |
| **Familiar, high-attractiveness faces** | | | | | |
| High | 0.153 | 0.113 | -7.220 | -0.097*** | -0.863 |
| Low | 0.110 | 0.109 | -10.725 | -0.140*** | -1.282 |
| Average | 0.279 | 0.183 | 1.304 | 0.029 | 0.156 |
| Self | 0.459 | 0.168 | 10.381 | 0.209*** | 1.241 |
| **Familiar, low-attractiveness faces** | | | | | |
| High | 0.104 | 0.107 | -11.403 | -0.146*** | -1.363 |
| Low | 0.120 | 0.121 | -8.981 | -0.130*** | -1.073 |
| Average | 0.281 | 0.202 | 1.304 | 0.031 | 0.156 |
| Self | 0.494 | 0.203 | 10.078 | 0.244*** | 1.241 |

*Note*. One sample Student t-test for chosen proportion of test face on unfamiliar/familiar condition with high/low attractiveness (Test value = 0.25, two-tailed). *Stands for *p* value = < .05, ***p* value = < .01, ****p* value = < .001.
